# A GC-MS Metabolic Study on Lipophilic Compounds in the Leaves of Common Wheat *Triticum aestivum* L.

**DOI:** 10.3390/metabo14080426

**Published:** 2024-08-01

**Authors:** Asya R. Vasilieva, Nikolay M. Slynko, Nikolay P. Goncharov, Ljudmila E. Tatarova, Leonid V. Kuibida, Sergey E. Peltek

**Affiliations:** 1 Federal Research Center, Institute of Cytology and Genetics, Siberian Branch of the Russian Academy of Sciences, Akademika Lavrentieva Avenue 10, 630090 Novosibirsk, Russia; nslynko@mail.ru (N.M.S.); gonch@bionet.nsc.ru (N.P.G.); ljudatat@mail.ru (L.E.T.); peltek@bionet.nsc.ru (S.E.P.); 2 Kurchatov Genomics Center, Institute of Cytology and Genetics, Siberian Branch of the Russian Academy of Science, Akademika Lavrentieva Avenue 10, 630090 Novosibirsk, Russia; 3 Institute of Chemical Kinetics and Combustion, Siberian Branch of Russian Academy of Sciences, 630090 Novosibirsk, Russia; kuibida@kinetics.nsc.ru

**Keywords:** *Triticum aestivum* L., metabolic profiling, GC-MS, GCxGC-MS, adaptation mechanisms, chemotaxonomy, octacosan-1-ol, fatty acid alkylamides

## Abstract

Common wheat (*Triticum aestivum* L.) is one of the most valuable cereal crops worldwide. This study examined leaf extracts of 30 accessions of *T. aestivum* and its subspecies using 48 h maceration with methanol by GC-MS and GCxGC-MS. The plants were grown from seeds of the wheat genetics collection of the Wheat Genetics Sector of the Institute of Cytology and Genetics, SB RAS. The analysis revealed 263 components of epicuticular waxes, including linear and branched alkanes, aliphatic alcohols, aldehydes, ketones, β-diketones, carboxylic acids and their derivatives, mono- and diterpenes, phytosterols, and tocopherols. Hierarchical cluster analysis and principal component analysis were used to identify and visualize the differences between the leaf extracts of different wheat cultivars. Three clusters were identified, with the leading components being (1) octacosan-1-ol, (2) esters of saturated and unsaturated alcohols, and (3) fatty acid alkylamides, which were found for the first time in plant extracts. The results highlight the importance of metabolic studies in understanding the adaptive mechanisms and increasing wheat resistance to stress factors. These are crucial for breeding new-generation cultivars with improved traits.

## 1. Introduction

Common wheat (*Triticum aestivum* L., genome BBAADD, 2n = 6x = 42) is one of the most valuable cereal crops in the world [1]. The number of its cultivars and subspecies is vast and continues to grow [2]. Studies focusing on the primary and secondary metabolism of wheat plants yield valuable insights into the phenotypic intraspecific diversity of *T. aestivum* [3]. Metabolic research is now becoming increasingly relevant due to the popularity of functional nutrition [4] and deep grain processing [5]. The dissimilarities found in the metabolic profiles of various types and endemic subspecies of common wheat may offer insights into their capacity to adapt to different environmental conditions, combat diseases and pests, and exhibit diverse characteristics in terms of qualitative and quantitative yield and grain quality [6]. In the context of agriculture, where the imperative to enhance wheat productivity is intensifying, an in-depth comprehension of these distinctions is of paramount importance [7]. Metabolic studies are fundamental to chemotaxonomic classification and identification of organisms based on differences in their chemical composition, including their secondary metabolites [8]. Such knowledge can be used in a wide range of applications. These include the breeding of new varieties with desirable characteristics, such as improved grain quality, increased resistance to stress conditions, diseases, and pests, and better adaptation to local and global climate change [9].

Plants are organisms with a large functional surface area interacting with the environment. Hence, the surface wax layers of vegetative parts, such as stems and leaves, are crucial for adaptation. In the vast majority of cases, this boundary layer is a hydrophobic cuticle. The cuticle has a morphologically mixed structure: a mechanically tough matrix (cutin, cutane, polysaccharides) that is insoluble in organic solvents, and organically soluble compounds called wax. Intra- and epicuticular waxes (EWs) are distinguished, and are a vast group of chemical compounds [10]. Intracuticular waxes are deposited in a matrix of the mechanically stable polymer cutin, forming a proper cuticle layer. EWs are deposited on the surface of this layer, with a mixture of lipids and polysaccharides (cellulose and pectin) located below the cuticle layer. EWs may appear as a smooth and transparent layer or as a dull gray plaque. These waxes are composed of crystals of various shapes, including plates with regular or irregular edges, rods, threads, tubes, and others. The chemical composition of EWs (lipophilic metabolites) commonly comprises very-long-chain compounds, namely fatty acids, their derivatives, primary and secondary alcohols, esters, aldehydes, alkanes, and ketones. Also, many types of waxes comprise cyclic compounds, triterpenoids, flavonoids, and sterols [10].

Mass spectrometry is a reliable technique for metabolic investigations, enabling the precise identification and quantification of metabolites in plant samples. Additionally, it offers a comprehensive analysis of metabolic pathways and their modifications in relation to both external and intrinsic factors [11,12,13,14].

This study aims to determine the chemical composition of lipophilic metabolites in wheat leaf extracts by employing mass spectrometry. The anticipated results will enable the exploration of relationships within individual metabolic pathways and between these specific pathways. This comprehensive study aims to analyze a diverse range of common wheat varieties and subspecies.

## 2. Materials and Methods

### 2.1. Plant Material

This study investigated a collection of 30 accessions, including 23 accessions of common wheat (*T. aestivum*), 4 of its subspecies (*T. aestivum* ssp. *petropavlovskyi* (Udacz. et Migusch.) N. P. Gontsch., *T. aestivum* ssp. *tibetanum* J. Z. Shao and *T. aestivum* ssp. *yunnanense* King ex S. L. Chen, 1 tetra- (*T. cartlicum* Nevski)), and 2 of diploid wheat (*T. monococcum* L.), from the collection of the Wheat Genetics Sector of the Institute of Cytology and Genetics, SB RAS (Table S1). The plants were grown in the field at the Institute’s experimental plot. The sampling process was strictly confined to the period from 11:00 AM to 12:00 PM on 30 June 2023. In order to calculate the average leaf arrangement for each cultivar, a single mean was selected from a sample of 5 plants. Fragments weighing 200 mg were extracted from individual leaves. Subsequently, fragments from all leaves (samples) were pooled for each cultivar, resulting in a total weight of 1000 mg. The extraction of lipophilic metabolites commenced on the same day as the accessions.

A preliminary experimental study was conducted to investigate the potential for oxidation of certain compounds during the extraction process. The results indicated that aliphatic aldehydes, which are particularly susceptible to oxidation, were present in significant quantities in the extracts. However, their corresponding oxidized forms, acids, were not detected in the same extracts. In light of this observation, the decision was taken not to add antioxidants to the working extracts of wheat leaves. The final results of the aldehyde-to-acid ratio are presented in Table S2. The nomenclature of all the mentioned below compounds, as designated by IUPAC, is provided in Table S2, along with their trivial names.

### 2.2. Reagents and Solvents, Synthesis of Pure Substances

All chemical reagents and solvents (extra pure for GS-MS) were purchased from Sigma-Aldrich, Inc. (Steinheim, Germany). Lupeol used as a standard was kindly provided by the Department of Medicinal Chemistry, N. N. Vorozhtsov Institute of Organic Chemistry (Novosibirsk, Russia). The hydrocarbon mixture of standard quality was acquired from Agilent Technologies (North Kingstown, RI, USA). A standard mixture of fatty acid methyl esters was obtained from Sigma-Aldrich (Steinheim, Germany). Benzaldehyde (ReagentPlus, CAS 100-52-7), benzoic acid (For analysis, CAS 65-85-0), acetophenone (ReagentPlus, CAS 98-86-2), hydroquinone (Technipur, CAS 123-31-9), trans-ferulic acid (matrix substance for MALDI-MS, CAS 537-98-4), palmitic acid (≥99%, CAS 57-10-3), and stearic acid (≥98.5%, CAS 57-11-4), also obtained from Sigma-Aldrich, were used as chromatographic standards. Dimethylamine (40% aqueous solution for synthesis, CAS 124-40-3), ethylamine (66.0-72.0% aqueous solution for synthesis, CAS 75-04-7), and thionyl chloride (for synthesis CAS 7719-09-7) were used for fatty acid amide synthesis. Methanol (≥99.9%, CAS 67-56-1) was used for extractions.

#### Synthesis of Fatty Acid Amides

Fatty acid amides were synthesized following the procedure detailed by Loscher et al. in 2015 [15]. Palmitic and stearic acids were first converted into their acyl chlorides and then reacted with dimethylamine and ethylamine. The melting point of N,N-dimethylhexadecanamide was found to be in the range of 39.5–40.5 °C (lit. 41 °C [16]). For N,N-dimethyloctadecanamide, the melting point range was between 50 and 51.5 °C (lit. 50.5–51.5 °C [16]). For N-ethylhexadecanamide, it was 72 °C (lit. 70–71.5 °C [17]), and for N-ethyloctadecanamide, it was 77–78 °C (lit. 78 °C [17]). The NMR 1H spectral data of the synthesized amides were found to be identical to those reported in the literature [15].

### 2.3. Extraction of Metabolites

The determination of extractives involved macerating 1000 mg of the prepared material with 10 mL of methanol in a flask at room temperature. The mixture was left to stand and occasionally stirred for 48 h. A Schott glass filter with a pore size of 10–16 μm was used to filter the extract from each sample. An injection of 1 µL of the prepared extract from each sample was made into the injector of both an Agilent Technologies 6890 gas chromatograph and an Agilent Technologies 7890B gas chromatograph.

### 2.4. Chromatographic Separation and Mass Spectrometric Detection

The annotation of compounds was performed using two systems: the Pegasus 4D GCxGC-TOF MS (which incorporates the Agilent Technologies 7890B) and the Agilent Technologies 6890 chromatograph with 5973 mass spectrophotometric detector. The data from the Agilent Technologies 6890 chromatograph with 5973 mass spectrophotometric detector were used to further verify the compounds identified on the Pegasus 4D GCxGC-TOF MS.

#### 2.4.1. GC-MS Detection

The analysis conditions in one-dimensional mode on Agilent Technologies 6890 were as follows. The analysis conditions in one-dimensional mode on Agilent Technologies 6890 were as follows’, the sentence ‘GC-MS procedure described [18]’ is inserted. The RIs were then calculated based on the peak retention times, using the approach outlined by Dool and Kratz [19]. Peaks were integrated using the scanning mode with total ion current measurement in the mass range of 10–800 amu and the selective ion scanning mode corresponding to the *m*/*z* value of the characteristic ion with the highest intensity (Q_m_). The Xcalibur 2.0 program was used to calculate the peak areas of all components. Identification of the components involved the utilization of the NIST Mass Spectral Search Program for the NIST/EPA/NIH Mass Spectral Library Version 2.4, as well as comparing the obtained RI values with the extended version of the same database [20].

#### 2.4.2. GCxGC-MS Detection

The samples were analyzed in two-dimensional mode using Pegasus 4D GCxGC-TOF MS under the conditions shown in Table 1.

### 2.5. Data Processing and Annotation

The chromatograms were processed using LECO ChromaTOF software, which performs automatic searches and comparisons with NIST electronic databases [20]. As the nomenclature of compounds annotated by LECO ChromaTOF does not always correspond to the IUPAC standard, the names are provided in the text and in Table S2 in accordance with the requirements of this nomenclature (with the possible addition of trivial names).

Table S2 displays the average values of the ratios between the peak areas of the identified compounds and the total peak areas, expressed as percentages. These ratios were calculated by referencing the peak areas of the internal standard.

### 2.6. Statistical Analysis

The technique of hierarchical cluster analysis was employed to visually depict the variations among the investigated leaf extracts of various wheat cultivars. In [18], data combined by metabolic pathways were taken for statistical analysis. In this work, such an approach was not implemented due to the apparent predominance of a small number of metabolic pathways over the others. We applied multivariate analysis approaches, particularly the principal component analysis (PCA), for more comprehensive coverage and deeper analysis of the processes under study. All compounds listed in Table S2 were included as variables in the principal coordinate analysis (PCA) and unweighted paired group method with arithmetic mean (UPGMA). The PCA processing results provided the eigenvalues and variation percentages for the first principal components (PC1 and PC2), which accounted for 48.94% of the total sample variance. The evaluation of similarity indices was conducted using the Bray–Curtis method.

Multivariate analysis was conducted using the software Past 4, version 4.11 (Hammer et al., 2001 [21]).

## 3. Results

**Figure 1:**
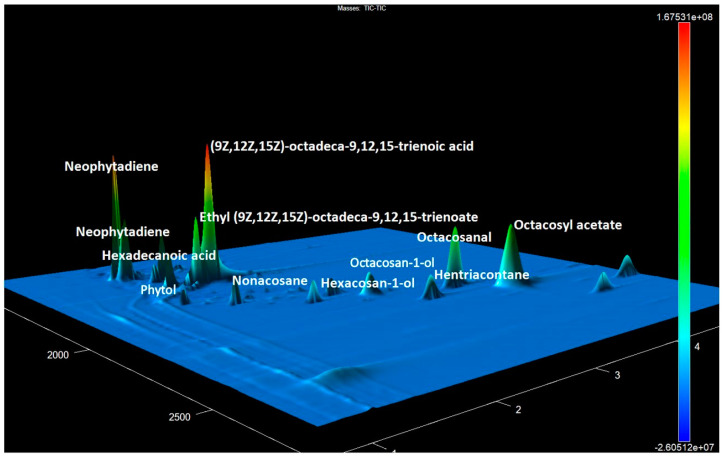
The GCxGC-TOF MS chromatogram fragment of accession No. 15 contains the most prevalent compounds of various classes.

The data interpretation based on mass spectra analysis demonstrated that aliphatic alcohols of linear structure and their derivatives had the highest abundance in most of the extracts (Table S2) (Figure 1).

**Figure 2:**
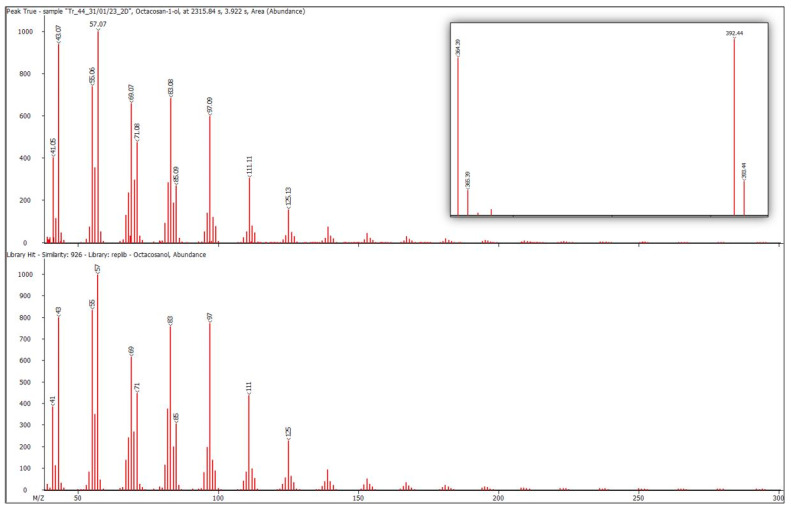
The mass spectrum of the octacosan-1-ol peak from the chromatogram of accession No. 44 (**top**) and the library mass spectrum of octacosan-1-ol (**bottom**) are presented. The convergence was 926. The inset on the right is an enlarged part of the mass spectrum with characteristic *m*/*z* 364 and 392.

The main compounds involved octacosan-1-ol, triacontan-1-ol, and octacosyl acetate. The mass spectrum of octacosan-1-ol exhibited distinct signals at *m*/*z* 57, 69, 83, and 97, along with 364 (M^+^-46) and 392, resulting from water detachment from the molecular ion (Figure 2). The molecular ion, which had a mass-to-charge ratio of 410, was almost undetectable. The mass spectrum of octacosyl acetate was completely indistinguishable from the mass spectrum of octacosan-1-ol, with the production of the *m*/*z* 392 ion resulting from acetic acid cleavage. The ions specific to tetracosan-1-ol had *m*/*z* values of 364 and 336, whereas those of hexacosan-1-ol had *m*/*z* values of 336 and 308.

The most important hydrocarbons in the C10–C40 range were found to be linear and branched alkanes. The structural confirmation of linear alkanes relies on several factors, including the predominant peak at *m*/*z* 57, the characteristic decrease in peak intensities from *m*/*z* 57 to 127, the appearance of low-intensity molecular ion peaks, and the consistency of the Kovacs index (RI). Furthermore, the structure of linear alkanes was accurately determined by employing the programs Xcalibur 2.0 AMDIS and LECO ChromaTOF, corroborated by the matching mass spectra of standard sample compounds. Almost all branched alkanes were also found to exhibit the most intense peak at *m*/*z* 57, with the distribution of peak intensities in the region of *m*/*z* 57–127 unlike linear structures and the mass spectra possibly exhibiting characteristic peaks with *m*/*z* [M-CH_3_]^+^, [M-C_2_H_5_]^+^, and [M-C_3_H_7_]^+^. In case cleavage occurs within the chain depth, the mass spectrum will exhibit ion signals with *m*/*z* values corresponding to the length of the resulting fragments [22]. In case the mass spectra of the determined hydrocarbons were not available in the NIST database, their structure was determined according to the general cleavage patterns described in [23]. A total of 25 alkanes with linear structures and 42 alkanes with branched carbon chain structures were determined.

The structure of carboxylic acids and their derivatives, excluding N-ethylamides, was determined by comparing mass spectra using the AMDIS program and LECO ChromaTOF, in conjunction with referencing literature data. A total of sixteen saturated aliphatic acids with their five esters, five unsaturated acids with their six esters, and two lactones were identified.

**Figure 3:**
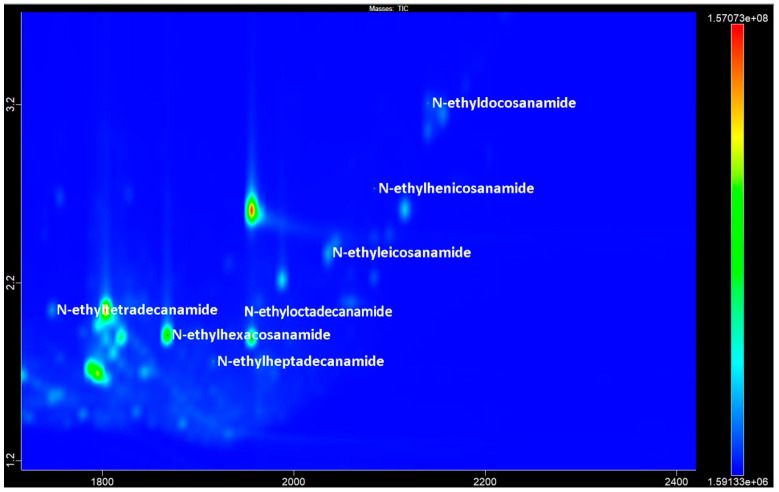
The GCxGC-TOF MS chromatogram fragment of accession No. 1 contains the most prevalent compounds of the fatty acids N-ethylamides.

The peaks in mass spectra with RIs of 2043, 2267, 2367, 2467, 2667, 2767, and 2863 by the AMDIS LECO ChromaTOF program were defined as either N-ethylamides or N,N-dimethylamides of tetradecanoic, hexadecanoic, heptadecanoic, octadecanoic, eicosanoic, henicosanoic, and docosanoic acids, respectively (Figure 3). A view of the chromatogram of the same accession obtained by GC-MS is shown in Figure S1.

The mass spectra of all these compounds exhibit peaks at *m*/*z* 72, 87, and 100, with RIs confirming their classification within the homologous series. In order to provide clarity regarding this classification, we synthesized both N-ethyl and N,N-dimethyl palmitic acid and octadecanoic acid amides. When these compounds were used as internal standards for co-injection, the peak in the mass spectrum of the wheat leaf extract with RI 2267 matched that of N-ethylpalmitamide, as did the peak with RI 2467 with that of N-ethylstearamide, whereas the peaks of N,N-dimethylpalmitamide and N,N-dimethylstearamide had RI values of 2206 and 2407, respectively. (Z)-13-Docosenamide was also identified in the extracts of some accessions (Table S2).

Aliphatic aldehydes and ketones are represented by 14 linear saturated and 5 unsaturated aldehydes, four linear and two branched ketones, and 4-hydroxy-2-butanone, as well as three β- and one γ-diketones. Although the mass spectrum analysis fails to reliably assign the position of the hydroxyl group in the compound hydroxy-14,16-hentriacontanedione, the position of the hydroxyl group was attributed to the C8 or C9 atoms in [24]. Furthermore, within the extracts of certain accessions, the presence of 1,1-dibutoxybutane, a diacetal derivative of butyraldehyde, was detected. Included in this category are compounds such as 2,6,6-trimethyl-1-cyclohexene-1-acetaldehyde, 1-(3,6,6-trimethyl-1,6,7,7a-tetrahydrocyclopenta[c]pyran-1-yl)ethanone, trans-β-Ionone, 3-hydroxy-5,6-epoxy-β-ionone, and 4-hydroxy-β-ionone. These compounds are produced in plants due to the cleavage of carotenoids [25].

Isoprene derivatives include monoterpenes (11) and their plant-derived cymenes (2), diterpenes (17), sesquiterpenes (1), steroids and their derivatives (13), and their precursors (2). Additionally, this group encompasses compounds containing a polyprenoid fragment in their structure. These include (2R)-2,5,7,8-tetramethyl-2-[(4R,8R)-4,8,12-trimethyltridecyl]-3,4-dihydrochromen-6-ol (vitamin E), tocopherol γ, their esters, tocospiro A and B, and 4,8,12,16-tetramethylheptadecan-4-olide. In addition, the chromatograms revealed Geranylacetone, Farnesylacetone, and Geranyllinalool.

Aromatic compounds include phenols (13) and their derivatives (5), benzoic acid and its derivatives (3), aromatic systems conjugated with a carbonyl group (5) or with a double bond (3), and condensed systems (5). In addition, the chromatograms revealed 2-phenylacetaldehyde.

Besides the above, the analysis identified five alkenes, 2-pentyl-furan, dihydroactinidiolide, loliolide, and (5Z)-2,6,10-trimethylundeca-1,5,9-triene.

## 4. Discussion

The EW films are of significant importance in the regulation of cuticular water transport in plants, which is essential for their survival [26]. In addition, these films act as protective barriers against microorganisms, including fungi and bacteria [27]. Different types of EW films can be found in nature, each having unique chemical compositions and structures [28]. Typically, EW films are composed of a combination of C20-C40 *n*-alcohols, *n*-aldehydes, very-long-chain fatty acids, and alkanes [28,29]. The thickness of these films can range from nanometers to micrometers, exhibiting a crystalline structure [29]. The surface morphology of plant EW films is characterized by porosity [30] and hydrophobicity. It is believed that these properties contribute to the water permeability of the cuticle. Powerful antioxidants that help protect cell membranes from oxidative damage caused by free radicals and reactive oxygen species include phytol, its isomers and esters, tocopherols, and polyunsaturated fatty acids. This activity is particularly important for the leaf epicuticle, which is constantly exposed to ultraviolet radiation and other external stressors that promote the formation of free radicals. In addition, these compounds can absorb UV radiation, thereby preventing its penetration into the deeper layers of the leaf, where it can damage photosynthetic complexes and DNA. This characteristic enhances the resistance of plants to UV stress [31].

The composition of EWs in wheat leaves was first studied using GC-MS [32,33]. These studies revealed the presence of aliphatic alcohols with dominant octacosan-1-ol, hydrocarbons, wax esters, β-diketones, and hydroxy analogs in the composition of EW.

Later works [34,35,36,37] generally confirmed the results but also added aliphatic aldehydes to the list of identified classes of compounds. Given that these studies were conducted on a small number of varieties using different sampling conditions, sample preparation and analysis techniques, different instrumentation, and different programs for processing research results, it is difficult to search for common patterns when analyzing the data obtained.

### 4.1. Aliphatic Alcohols

According to [35,37], the octacosan-1-ol content in wheat cuticular wax extracts is indicative of the presence of metabolic polymorphism. The presence of primary alcohols and octacosan-1-ol in the cuticle is associated with the formation of lamellar-type crystals [30], which provide the plant with resistance to drought and high temperatures and are associated with plant-pathogen interactions. (E,7R,11R)-3,7,11,15-tetramethylhexadec-2-en-1-ol (phytol) and its isomers perform a similar function in cuticular waxes, but their structures differ from those of alcohols by the presence of a branched hydrocarbon chain and double bonds. The content of alcohols in [34] was estimated to range from 40% to 70% in the wheat samples tested, and in our study, the extracts were found to contain alcohols and their esters in the range from 0.13% to 48.77%. For example, the content of alcohols was identified to be up to 2.36% in common wheat varieties (accession Nos. 1, 8, 16) cv. Baganochka (Novosibirsk region, Russia), K-30947 (Tyva, Russia), ssp. *lutinflatum* k-39218 (Sichuan, China), respectively, with the highest values (more than 30%) found in accession Nos. 7, 10, 17, 15, namely landraces K-24849 from Yakutia and K-28628 from China, KU 221-21 from Japan and KU510 from China. In addition to primary alcohols, secondary alcohols were detected in the works, for example, in [38]. The content of secondary alcohols in the extracts of our accessions was found to be relatively low, not exceeding 0.79% in total (in accession No. 19, Australian common wheat variety Dirk, AUS 90054).

### 4.2. Esters of Aliphatic Alcohols

Octacosan-1-ol in the form of an ester with acetic acid was detected in the extracts of accession Nos. 2, 6, 13, 15, 17, and 9 (line 278/9 of Novosibirsk; *T. aestivum, lutescens* K-59580; cv. Tulun 15 of Irkutsk region K-64599, *T. petropavlovskyi* K-43351 from China, *T. yunnanense* KU510, wheat KU 221-21 from Japan, landrace K-8009 (Mongolia, Umnegov)). In leaf extracts of all these accessions except No. 9, octacosan-1-ol was the predominant compound. A similar result was reported in [39]. Octacosyl acetate exhibits higher hydrophobic properties than octacosan-1-ol itself, allowing the formation of a water-repellent layer on the plant surface to prevent excessive wetting and facilitate water outflow. The formation of octacosyl acetate in the plant is similar to the reactions of wax ester formation and proceeds with the help of enzymes that belong to the group of acyltransferases. One of the key enzyme systems involved in this process is acyl-CoA/alcohol acyltransferases (AWATs or WSD1). These enzymes catalyze the transfer of the acyl group from acyl-CoA to alcohol, resulting in the formation of aliphatic alcohol acetate. The same is the case with phytyl esters, which constitute a significant proportion in the extracts of accession Nos. 14 and 24 (KU515 endemic Chinese hexaploid wheat *T. tibetanum* (Tibet Autonomous Region, China) and tetraploid wheat *T. carhtlicum* k-14940 (Georgia)).

### 4.3. Alkanes

The qualitative composition of cuticle alkanes in cereals has been extensively studied and reported in numerous works, including [33,34,35,37,40]. At the same time, a number of studies revealed only linear alkanes with the number of C atoms being 23–35. It is a commonly observed phenomenon that alkanes with an odd number of carbon atoms dominate over alkanes with an even number of carbon atoms, which is a defining property of the cereal cuticle composition [40].

Our research focused on analyzing the alkane composition in leaf extracts from thirty different wheat cultivars. Nonacosane was the most frequently found compound in 14 of the extracts, whereas hentriacontane was the predominant compound in 8 of the extracts. The predominance of these hydrocarbons (C29, C31) is not uncommon for cereals. However, an extensive chromatographic study of leaf extracts of this family has provided examples out of the above range. *Alopecurus gerardi* has a maximum content of C25 (17.6%), while *Echinochloa crusgalli* (L.) has a maximum content of C27 (19.1%). In *Digitaria sanguinalis*, the maximum content is C35 (38.1%), and *Elymus giganteus* is primarily composed of an alkane with an even number of carbon atoms, specifically C34 (86.2%). Our study also revealed some dropouts from the general rule. In the extracts of four varieties, the predominant linear alkane was found to be pentacosane (accession Nos. 12, 16, 20, 21: *T. petropavlovskyi* K-44126 from China, *T. aestivum*, ssp. *lutinflatum* K-39218, *T. aestivum, lutescens* K-40329 Saratovskaya-210). In the extracts of two varieties, it was tricosane (accession Nos. 1 (cv. Baganochka) and No. 8 (K-30947). In the other two varieties, it was heptacosane (accession Nos. 13 *T. petropavlovskyi* K-43351 and No.14 KU515). According to the literature, the C29/C31 ratio changes during plant growth, with an increased presence of C31 during leaf senescence [36].

Another taxonomic parameter to take into account is the carbon preference index (CPI), which measures the ratio of odd-n-alkane homologs to even-n-alkanes. It is customary for plants that the abundance of odd-numbered alkanes, primarily derived from the metabolic decarboxylation of fatty acids with an even number of carbon atoms, should naturally surpass the abundance of even-numbered alkanes. It can be seen that the CPI values calculated for the alkane components of the extraction mixtures fell within the range of 0.79–24.37. It is notable that some of the extract accessions concentrated in the region of values 0.79–2.33, while others concentrated in the region 13.6–24.37. No correlation was identified between the CPI values obtained and the PCA characteristics.

The extracts of our samples demonstrated variation in the ratio of linear alkanes to branched alkanes, falling within three ranges. In the lower range, ranging from 1.75 to 3.90, there was a significant amount of branched compounds. The upper range consists of linear alkanes with ratios ranging from 8.08 to 19.46. In a comprehensive study of the EW composition of numerous cereals, similar results were obtained [41]. This analysis indicated a substantial occurrence of branched alkanes in the low range of ratios found for *Phleum bertoldi* (0.68), *Bromus catharticus* (1.08), *Phalaris canariensis* (1.79), and *Avena versicular* (1.37). At the same time, the predominance of linear alkanes was noted for *Elymus giganteus* (142), *Stipa calamagrostis* (28.4), and *Festuca pratentis* (7.13) [41].

The presence of branched alkanes in the cuticle, with a lower melting point compared to linear alkanes, is likely to enhance the flexibility of cuticle components, influencing the permeability of the leaf surface and the intensity of gas exchange. The process of metabolic synthesis of branched alkanes has been described in detail using *Arabidopsis thaliana* as an example [42]. This study suggests that valine, leucine, and isoleucine derivatives play a crucial role in creating chain branching in alkanes. Valine derivatives result in even iso-type branching, leucine derivatives lead to odd iso-type branching, and isoleucine derivatives contribute to even anteiso-type branching. The metabolic synthesis of alkyl malonate allows for branching in the depth of the hydrocarbon chain.

### 4.4. Fatty Acids

In accordance with [43], a considerable number of plant cuticular waxes are composed of wax esters with high melting points and fatty acids with exceptionally long carbon chains. The presence of very-long-chain fatty acids in cuticle composition has been reported in [29]. In the current study, we identified saturated fatty acids with carbon chains of C16, C18, C21, and C22, as well as unsaturated fatty acids with carbon chains of C16 and C18. Additionally, we detected esters derived from these fatty acids. Among these, we identified methyl, ethyl, and phenylethyl esters. The methyl esters may have formed as a result of esterification during sample preparation. However, the presence of ethyl esters in all cases is at least as high as that of methyl esters, suggesting that the role of esterification in the formation of methyl esters in the samples can be discounted. Many plants synthesize phenylethyl esters as constituents of their essential oils, which act as attractants for insects. The potential purpose of the intermediate formation of fatty acid phenylethyl esters might be to facilitate the transportation of bound phenylethanol to the vaporization zone. These compounds play a crucial role in the development of the lipophilic layer of cell membranes and the creation of wax esters.

### 4.5. β-Diketones and Their Hydroxy Derivatives

A very important class of cuticle compounds is β-diketones and their hydroxy derivatives [35,44,45]. The presence of β-diketones in wheat was initially observed in 1969 [24]. Subsequent investigation revealed that the occurrence of β-diketones and their hydroxy derivatives in the epicuticular layer of wheat alters the optical characteristics of this layer, resulting in the manifestation of a glaucous hue on the surface of stems and leaves [46]. The works of the following years revealed the relationship between the presence of β-diketones and the tubular shape of crystals of cuticular waxes [35]. The results of the correlation analysis conducted in [36] indicate a direct correlation between glaucousness level and plant protection in the presence of drought.

**Figure 4:**
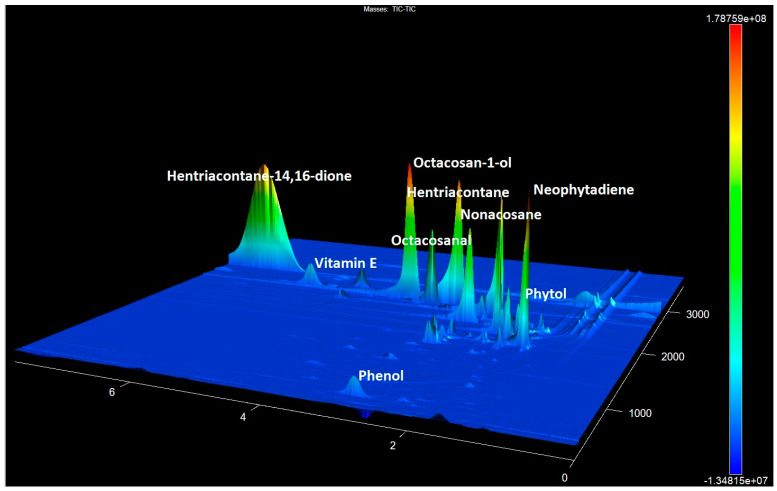
The GCxGC-TOF MS chromatogram fragment of accession No. 25 contains the predominant 14,16-hentriacontanedione and other components with a high abundance.

The content of β- and OH-β-diketones in the identified compounds for glaucous cultivars was observed to vary between 41.0% and 69.6% in [36]. The content of β- and OH- β-diketones in our extracts exhibited a range of variations. This variation encompassed samples with no detectable signals, as well as samples with contents ranging from 1% to 9.15% and samples with contents ranging from 11.82% to 29.4% (Figure 4) [47].

### 4.6. Other Compounds of Signaling Systems Functioning in Leaves

Among all annotated compounds, those with significant volatility, conventionally assigned to compounds with a maximum of 16 carbon atoms, play a variety of roles. This separation may be related to the presence of a distinct fatty acid biosynthesis pathway up to C16 occurring in plant plastids. Subsequently, the products can undergo additional modifications into fatty acids (FAs) and very-long-chain fatty acids (VLCFAs) or be subjected to biosynthesis for the production of other compounds. This category encompasses compounds belonging to different metabolic classes, including hydrocarbons, aldehydes, ketones, carboxylic acids, and their esters. All these compounds are integral to the signaling systems functioning in leaves. Specifically, when it comes to defense against pests and pathogens, styrenes, like other aromatic compounds, exhibit potential repellent or toxic properties that can deter insects or fungi. It has been observed that the infection of wheat leaves with yellow rust leads to the production of phenolic compounds, which are known to have natural fungicidal properties [48]. In the extracts of the accessions studied in this investigation, the content of phenolic compounds was found to vary from a value of less than 1% (accession Nos. 1, 8, 28 (cv. Baganochka, K-30947, K-27655 (Canada)) to about 9% (accession Nos. 10, 20, and 26 (common wheat 6638 Witchi Wheat (North Gansu, China), *T. aestivum, lutescens* K-40329 Saratovskaya-210), *T. monococcum* Mute Sog1 KT3-25 from Czech Republic)). These results may indicate the presence of either developed or natural resistance to this pathogen. Another example is the presence of 6-methoxy-2-benzoxazolinone in some extracts (accession Nos. 13, 14 (*T. petropavlovskyi* K-43351 and *T. tibetanum* KU515), which is an egg-laying stimulant for Hessian flies [49]. Numerous phenols serve as construction materials for the formation of the lignin framework.

### 4.7. Fatty Acid Amides

It is worth highlighting the discovery of a homologous series of saturated fatty acid amides from C14 to C24 in specific sample extracts (Table S2), which are predominantly composed of acids with an even number of carbon atoms. The presence of fatty acid amides, with a total content exceeding 1%, was observed in six extracts. The plant material from which they were obtained originated from disparate geographical zones (Table S1). The literature provides evidence of carboxylic acid amide derivatives being present in plant extracts [50]. A significant proportion of the amide derivatives documented in the literature exhibit the presence of one or more unsaturated groups in the acidic segment. Some saturated hydrocarbon derivatives can have the N-alkyl component represented by isobutylamine, tyramine, pyrrolidine, and other compounds [51,52,53,54]. The amides found in the samples, along with their corresponding N-ethylamides, are the result of decarboxylation of biogenic amino acids. The metabolic synthesis of fatty acid N-ethylamides remains undocumented in known metabolic databases. It can be postulated that this process encompasses the following stages: (1) the interaction between the fatty acid corresponding to the future amide and acetyl-CoA, accompanied by ATP consumption; (2) the activated acid reaction with alanine, resulting in the formation of the fatty acid N-acylalanine; (3) the reaction involving the decarboxylase enzyme and pyridoxal-5′-phosphate, leading to the generation of N-ethylamide [55]. Given that the content of these compounds in wheat extracts was found to be 46.09% (accession No. 8 of the common wheat landrace K-30947), it is plausible to suggest that this class of compounds might have a significant impact on plant adaptation to environmental variations. However, as noted in [38,56], “the actual contribution of different components of the cuticle to each of its functions remains unclear,” and the contribution of fatty acid amides to the adaptation process also remains unclear. The higher melting point displayed by N-ethylamides of fatty acids, compared to their corresponding free fatty acids [17], may indicate their potential role in influencing the plasticity of the EW.

### 4.8. Aliphatic Aldehydes

The range of long-chain aldehyde content in this study was observed to be between 0.27% and 4.45%. The group with minimum content (less than 1%) includes accession Nos. 8 and 16 (K-30947), Chinese endemic common wheat subspecies *T. aestivum* ssp. *lutinflatum* k-39218). The group with maximum content (more than 4%) covers accession Nos. 26 and 9 (*Sog1* mutant of *T. monococcum* (KT3-25) and common wheat landrace K-8009). Various plant signaling systems incorporate low-molecular-weight aldehydes and ketones, with a content not exceeding 0.5%.

## 5. Statistical Processing Findings

**Figure 5:**
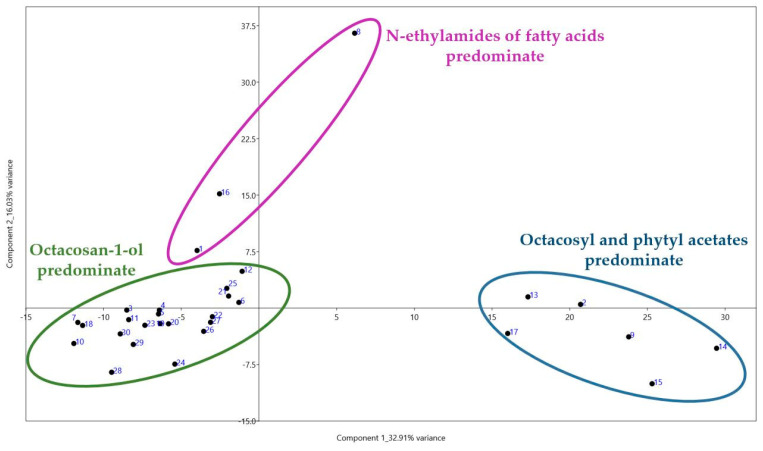
Biplots of PCA based on all identified compounds in 30 wheat varieties.

PCA calculated from the data of all compounds identified allowed us to distinguish several clusters with possible chemotaxonomic similarity (Figure 5). The samples that exhibit a significant presence of octacosan-1-ol can be distinguished as a separate cluster within the negative PC1 value zone, similar to samples dominated by octacosyl and phytyl acetates. The samples characterized by a prevalent occurrence of fatty acid alkylamides are placed in a separate section.

Analysis of compounds present in samples from both the positive and negative zones suggests that acylation of hydrophilic fragments of aliphatic alcohols is a response to changes in the water content of plant leaves. As a result, the cuticle gains hydrophobic properties, effectively hindering the infiltration of excess moisture into the leaf.

**Figure 6:**
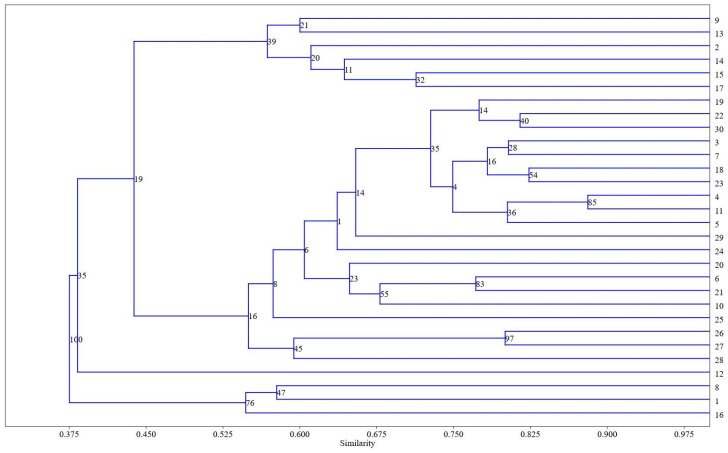
Dendrogram of hierarchical clustering for all identified components of extracts of 30 wheat varieties. Similarity indices are plotted on the Y-axis, and accession identification numbers are plotted on the X-axis.

Hierarchical cluster analysis was chosen to visualize the differences between the studied extracts of wheat varieties. Figure 6 displays the dendrogram of the lipophilic components of the extracts, as determined by UPGMA (unweighted paired group method with arithmetic mean).

The coefficient correlation coefficient of 0.8472 provides evidence that the dendrogram reliably reflects the similarity among the observations. The dendrogram illustrates the categorization into three primary branches. In the first branch, accessions Nos. 8, 1, and 16 (common wheat K-30947, cv. Baganochka, Chinese endemic common wheat subspecies *T. aestivum* ssp. *lutinflatum* k-39218), are dominated by metabolites with the highest content of fatty acid amides, with a similarity index of 0.550 estimated by the Bray–Curtis method and a bootstrap value of 76%. In the second branch, the accessions Nos. 13, 2, 14, 15, 17 (on the up), are dominated by alcohol acetates with a similarity index of 0.575, estimated by the Bray–Curtis method, with a bootstrap value of 39%. Accession No. 9 (landrace K-8009), whose extract is dominated by oleic acid, but whose octacosyl acetate content is slightly less, is also in the same cluster. All other accessions, including the most numerous group with octacosan-1-ol predominance, are located between the described groups.

## 6. Conclusions

- The differences observed in the composition of the extracts provide evidence for the existence of metabolic polymorphism in the studied *T. aestivum* varieties, which will allow breeding for these metabolites.
- The predominant component identified in the leaf extracts of most of the studied varieties was found to be octacosan-1-ol. Other markers of this polymorphism include N-ethylamides of fatty acids, acetates of octacosan-1-ol and phytol, and β-diketones.
- For the first time, N-ethylamides of fatty acids that may play an important role in plant adaptation to stress have been detected in plant extracts.
- No correlations were found between cuticle composition and the place of origin of wheat accessions.

## Figures and Tables

**Table 1:** GCxGC-MS analysis conditions on the Pegasus BT 4D system.

Injection	1 mkl, inlet split ratio 1:10, front inlet temp. 280 °C
Carrier gas	Helium, constant flow 1.4 mL/min
Column one	Rxi-5MS, 30 m × 0.25 mm i.d. × 0.25 µm coating (Restek)
Column two	Rxi-17Sil MS, 2 m × 0.25 mm i.d. × 0.25 µm coating (Restek)
Temp. program	50 °C (1 min); 5 °C/min to 150 °C: 10 °C/min to 250 °C: 20 °C/min to 280 °C (60 min): primary oven maintained +5 °C relative to secondary oven
Modulator timing	8 s; temp. maintained + 15 °C relative to secondary oven
Transfer line temp.	280 °C
Electron energy	70 eV
Ion source temp.	280 °C
Mass range (*m*/*z*)	40–650
Acquisition delay	300 s

## Data Availability

Data recorded in the current study are available in all Figures of the manuscript and in the Supplementary Materials.

## Supplementary Materials

The following supporting information can be downloaded at https://www.mdpi.com/article/10.3390/metabo14080426/s1. Table S1. List of studied accessions of common wheat. Table S2. Compounds identified in extracts from Triticum aestivum leaves by GC-MS. Figure S1. The GC-MS chromatogram fragment of accession No. 1.
