# Supplementary material for: A GC-MS Metabolic Study on Lipophilic Compounds in the Leaves of Common Wheat Triticum aestivum L
Source: Metabolites. 2024 Aug 1;14(8):426. doi: 10.3390/metabo14080426 (PMC11356221; doi:10.3390/metabo14080426)
Supplement: Supplementary file 1 [file metabolites-14-00426-s001.zip › Table_S1_S2_Fig_S1.pdf]

Table S1. List of studied accessions of common wheat.

№№	Cultivar	Voucher	Origin	Ploidy	Species, variety
01	Baganochka	K-67469	Novosibirsk region	2*n*=42	*T. aestivum*, var. *albidum*
02	Line 278/9	K-59580	Novosibirsk region	2*n*=42	*T. aestivum*, var. *lutescens*
03	Alenkaya Uimonskaya	K-23368	Altay region	2*n*=42	*T. aestivum*, var. *ferrugineum*
04	Kamchdalka	K-38586	Krasnoyarsk region	2*n*=42	*T. aestivum*, var. *milturum*
05	Sibirskaya	K-23347	Irkutsk region	2*n*=42	*T. aestivum*, var. *ferrugineum*
06	Tulun 15	K-64599	Irkutsk region	2*n*=42	*T. aestivum*, var. *lutescens*
07	Landrace	K-24849	Yakutsk region	2*n*=42	*T. aestivum*, var. *ferrugineum*
08	Landrace	K-30947	Тыва	2*n*=42	*T. aestivum*, population
9	Landrace	K-8009	Mongolia, Umnegovi	2*n*=42	*T. aestivum*,
10	6638 Witchi Wheat	K-28628	China, North Gansu	2*n*=42	*T. aestivum*,
11	Landrace	K-42183	China	2*n*=42	*T. aestivum*, var. *ferrugineum erythrospermum*,
12		K-44126	China	2*n*=42	*T. petropavlovskyi*
13		K-43351	China	2*n*=42	*T.petropavlovskyi*
14		KU515	China, Tibet	2*n*=42	*T. tibetanum*
15		KU510	China	2*n*=42	*T. yunnanense*
16	Landrace	K-39218	China, Sichuan	2*n*=42	*T. aestivum*, ssp. *lutinflatum*
17	*T. carthlicum*+*Ae. squarrosa*	KU 221-21	Japan	2*n*=42	amphiploid
18	NP 790	K-45749	India, Deli	2*n*=42	*T. aestivum*, var. *milturum*
19	Dirk	AUS 90054	Australia	2*n*=42	*T. aestivum*,
20	Saratovvskaya 210	K-40329	Saratov region	2*n*=42	*T. aestivum*, var. *lutescens*
21	Gremme		Tatarstan	2*n*=28	*T. dicoccum*
22	Riko	K-65588	Leningrad region	2*n*=42	*T. aestivum*, var. *erythrospermum*
23	Fori 5	K-65593	Leningrad region	2*n*=42	*T. aestivum*, var. *erythrospermum*
24	Landrace	K-14940	Georgia	2*n*=28	*T.carhtlicum*
25	*Vrn7*^{Sc}	BGI	former USSR	2*n*=42	*T. aestivum*
26	Mute *Sog1*	KT3-25	Czech	2*n*=14	*T.monococcum*
27	Landrace	PI355549	Austria, Vorarlberg	2*n*=14	*T.monococcum*
28	Prelude	K-27655	Canada	2*n*=42	*T. aestivum*, var. *hostianum*
29	Ciano F67	CIMMYT	Mexico	2*n*=42	*T. aestivum*,
30	Sieto Cerros 66	CIMMYT	Mexico	2*n*=42	*T. aestivum*,

All plants were grown in the experimental field at Institute of Cytology and Genetics, Novosibirsk, RF

Table S2. Compounds identified in extracts from *Triticum aestivum* leaves by GC-MS

Compound	1st Dimension Time (s)	2nd Dimension Time (s)	RI	Lit. RI	Q_m	Accessions																													
						01	02	03	04	05	06	07	08	09	10	11	12	13	14	15	16	17	18	19	20	21	22	23	24	25	26	27	28	29	30
Ethyl (*E*)-but-2-enoate	300	1,85	881	835	69					1,93				2,27	0,34	3,05		1,39	3,03		1,01	2,31				1,60			1,31						
4-Hydroxybutan-2-one	300	5,09	881		43		0,28	1,38	1,92	0,81				0,30		0,97	0,12	1,09	0,48	1,47		0,53		0,60	1,91	2,20	0,96		0,53	0,29				2,05	0,77
3-Hydroxy-5-oxooxolane-2,3-dicarboxylic acid	300	6,93	881		69					1,72								0,82																	
(*E*)-Hex-2-enal	308	2,01	886	851	41		0,27				0,25	0,12		0,29	0,06					0,71			0,35				0,44	0,30		0,02	0,08	0,23		0,52	0,48
3-Methylhexan-2-one	308	2,95	886	844	43		0,33	0,24	0,44	0,41		0,25											1,29	0,28		0,51	0,35				0,56				
1-Butoxybutane	316	5,92	891	858	57		2,98					0,27		0,28		0,42				4,15			3,56	0,36	1,99		0,14	3,49	0,39		0,51			1,74	1,51
Heptan-2-one	332	1,98	900	891	43						0,03	0,01	0,16						0,06	0,01		0,03	0,02				0,04	0,02			0,02				
Styrene	332	2,24	900	892	104	2,02	0,32	1,38	2,08			0,28		0,03	0,02		0,05	0,32		0,18	0,04	1,89	0,04		4,86	1,88	4,04	0,06	0,49	0,97	2,95	4,18		2,32	4,39

Oxolan-2-one	364	4,38	919	916	42	0,20	0,07	0,03	0,03	0,02	0,11	0,11	0,05	0,04		0,16	0,04	0,09	0,10	0,10	0,04	0,11	0,06	0,05	0,09	0,07	0,17	0,12	0,03	0,05	0,09	0,14		0,13	0,14
Benzaldehyde	436	3,27	962	962	77	0,06	0,13	0,02	0,02	0,05	0,17	0,28	0,06	0,13	0,10	0,19	0,05	0,22	0,21	0,25	0,07	0,10	0,15	0,26	0,21	0,10	0,23	0,12	0,10	0,07	0,29	0,23		0,14	0,16
Hexanoic acid	460	2,09	976	990	60	0,18	0,33	0,08	0,01	0,33	0,26	0,31	0,35	0,46	0,16	0,16	0,13	0,69	0,58	0,45	0,55	0,14	0,28	0,12	0,17	0,17	0,20	0,33	0,27	0,07	0,19	0,40		0,18	0,25
Phenol	468	2,88	981	981	94	0,18	1,76	2,74	2,86	1,26	4,73	3,55	0,58	1,69	5,40	5,40	1,13	3,29	3,60	2,67	1,13	3,19	2,91	4,47	8,77	5,15	3,93	2,27	2,28	3,52	8,72	5,08		2,65	4,56
Hexane-2,5-dione	476	2,19	986	921	43		0,07			0,04		0,06		0,04		0,06	0,01	0,13	0,07		0,04		0,03	0,08	0,03						0,07	0,05		0,04	
6-Methylhept-5-en-2-one	476	2,39	986	986	43	0,03	0,03	0,03			0,05	0,07	0,03	0,04	0,04	0,07	0,01	0,08	0,06	0,04	0,04		0,06					0,07	0,05	0,06	0,07				0,03
Prop-1-en-2-ylbenzene (α-Methylstyrene)	476	2,45	986	986	118	0,27		0,08	0,15			0,01				0,10	0,01	0,02		0,01		0,05	0,01	0,32	0,04	0,21	0,26			0,02	0,03	0,41		0,05	0,05
7-Methyl-3-methylideneocta-1,6-diene (β-Myrcene)	484	1,82	991	991	41		0,02					0,06		0,02	0,02	0,02		0,04	0,02	0,05		0,02		0,03		0,02	0,03					0,08			0,08
2-Pentylfuran	484	2,02	991	993	81		0,02							0,06				0,06	0,05	0,05		0,04					0,05								
2-Hydroxyoxolan-5-one	484	4,82	990	1011	57		0,05		0,17		0,05		0,09	0,02						0,07		0,03	0,08					0,09	0,13						
(*E*)-Hex-3-enoic acid	492	2,22	995	1003	41	0,15	0,34	0,04		0,13	0,21		0,15	0,23		0,25	0,05	0,26	0,24	0,52	0,16	0,07	0,30	0,11		0,19	0,33	0,39	0,20	0,20	0,44	0,82		0,39	0,35
(2*E*,4*E*)-Hepta-2,4-dienal	492	2,79	995	1012	81		0,08	0,01	0,01	0,02	0,03	0,17	0,07	0,15	0,08	0,09			0,08	0,07	0,04	0,01	0,08	0,04	0,03	0,02	0,01	0,11	0,10		0,03				0,12
Decane	500	1,50	1000	1000	57	0,01	0,09		0,02	0,03	0,02	0,29		0,11	0,08	0,08	0,01	0,18	0,04	0,05	0,02	0,06	0,14	0,05	0,01	0,04	0,03	0,08	0,07	0,01	0,02	0,61		0,05	0,02
2-Methyl-5-propan-2-ylbicyclo[3.1.0]hex-2-ene (3-Thujene)	500	1,98	1005	1005	93					0,02		0,30	0,01										0,08	0,12			0,16	0,08							
Octanal	500	2,23	1000	1003	43		0,03				0,03	0,12			0,07			0,04	0,02	0,08	0,03	0,04		0,03		0,03				0,02	0,08	0,07		0,04	
2-Methyl-5-propan-2-ylcyclohexa-1,3-diene (α--Phellandrene)	500	4,12	1000	1005	93																			0,26											
3,7,7-Trimethylbicyclo[4.1.0]hept-3-ene (3-Carene)	500	7,21	1005	1011	93		0,97			0,23	0,63	1,53	0,06	0,73	0,61	1,03	0,10	1,39	1,42	0,97	0,15	0,07	1,28	0,70			0,25	1,40	1,72	0,05	0,59	0,19		0,38	0,17
2,3,7-Trimethyloctane	524	1,53	1014		57	0,01	0,06			0,01	0,05	0,15	0,01	0,21	0,04			0,17	0,04	0,11		0,10	0,01	0,06	0,03	0,09	0,07		0,03		0,04	0,24			0,01
1-Methyl-2-propan-2-ylbenzene (o-Cymene)	532	7,38	1019		119					0,02											0,13								0,44	0,45					0,83
1-Methyl-4-propan-2-ylbenzene (p-Cymene)	540	2,24	1024	1025	119		0,12	0,01		0,01	0,21	0,34	0,02	0,15	0,16	0,26	0,05	0,34	0,15	0,27	0,02	0,40	0,08	0,51	0,24	0,29	0,42	0,06	0,06	0,02	0,36	0,68		0,11	0,15
1-Methyl-4-prop-1-en-2-ylcyclohexene (Limonene)	540	4,22	1024	1031	68		1,59		0,25	0,38	1,51			0,97	1,53	0,12	0,10	2,23	2,79		0,40	0,06	2,03	0,37	0,55	0,22		2,33	2,52	0,24	0,25	0,25		0,78	0,18
1,3,3-Trimethyl-2-oxabicyclo[2.2.2]octane (Eucalyptol)	548	2,00	1029	1032	68	0,09	0,33	0,51	0,95	0,07	0,67	1,12	0,04	0,34	0,29	1,47	0,08	0,61	0,37	0,54	0,04	0,59	0,34	0,86	0,71	0,58	1,31	0,38	0,22	0,08	0,67	1,04		0,45	0,40
2-Phenylacetaldehyde	572	3,65	1043	1045	91	0,17	0,21	0,04	0,03	0,20	0,16	0,26			0,16	0,08	0,01	0,20	0,36	0,15	0,13	0,07	0,26	0,12	0,13	0,03	0,16	0,40	0,19	0,07		0,09		0,13	0,13
Limonene isomer[1]	588	1,86	1052		68		0,01						0,01		0,02																				
5-Ethyloxolan-2-one	588	4,26	1052	1056	85	0,03	0,04			0,02	0,06	0,03	0,02	0,03	0,02	0,06	0,01	0,05	0,05	0,04	0,09		0,05	0,03	0,03	0,01	0,18	0,06		0,01	0,08	0,14		0,05	0,05
4-Methyldecane	596	1,47	1057		43			0,11	0,26		0,24				0,22			0,28		0,06				0,19		0,20	0,18			0,01		0,14			
1-Methyl-4-propan-2-ylcyclohexa-1,4-diene (γ-Terpinene)	596	2,14	1057	1060	93		0,02				0,04	0,04	0,15	0,02		0,04	0,01	0,05	0,03	0,03		0,02	0,02	0,04	0,03	0,01	0,04	0,02			0,04	0,04			
2-Methyldecane	604	1,50	1062	1064	57	0,04	0,02			0,05		0,10	0,02	0,12	0,51				0,10		0,08	0,13	0,01						0,06					0,12	
3-Methyldecane	612	1,56	1067	1071	57		0,01			0,01		0,02		0,03	0,03		0,78	0,05	0,02	0,01				0,03	0,01		0,02			0,01	0,03			0,01	0,01
3-Methylbenzaldehyde	612	3,53	1081	1071	91	0,04	0,01	0,03	0,03	0,02			0,01		0,01			0,01			0,02		0,01					0,02							0,01
1-Phenylethanone (Acetophenone)	612	3,67	1067	1066	105		0,06		0,01	0,03					0,16	0,21			0,13	0,08			0,12	0,26			0,20		0,09	0,21	0,47	0,21		0,10	0,26
Heptanoic acid	628	2,21	1076	1080	60	0,04	0,02		0,01	0,03		0,04	0,06	0,05	0,03		0,02	0,09	0,07		0,09		0,03					0,04	0,03						
(3*Z*,6*Z*)-Nona-3,6-dienal	660	2,69	1095	1100	67		0,02		0,01	0,03	0,03	0,02	0,01	0,03	0,02	0,02	0,01	0,04	0,03	0,02	0,02	0,01	0,02		0,02	0,01		0,03	0,03	0,01	0,01	0,02		0,01	0,02
Undecane	668	1,56	1100	1100	57	0,12	0,11	0,19	0,32	0,31	0,39	0,53	0,08	0,27	0,47	0,12	0,15	1,13	0,38	0,14	0,22	0,26	0,17	0,53		0,36	0,35	0,13	0,41	0,14	0,53	0,82			0,14
3,7-Dimethyloctan-3-ol	668	2,01	1100	1100	73							0,10		0,03		0,04		0,10		0,05														0,02	
Nonanal	676	2,36	1105	1104	82	0,01	0,09	0,03	0,03	0,15	0,16	0,13	0,03	0,08	0,08	0,07	0,03	0,06	0,10	0,09	0,04	0,03	0,11	0,05	0,02	0,06	0,06	0,08	0,07	0,04	0,07	0,08		0,10	0,05
3,4-Dimethylcyclohexan-1-ol	684	2,86	1110	1126	71	0,06	0,03			0,03		0,04	0,06		0,04			0,21					0,04			0,03	0,04			0,03	0,10	0,15			
2-Methyl-1-benzofuran	684	3,20	1110	1109	131		0,01				0,04	0,02		0,01	0,03	0,06		0,03	0,03	0,02		0,05	0,03	0,08	0,09	0,05	0,05	0,03		0,06	0,13	0,06		0,03	0,08
2,4-Dimethyldecane	692	1,55	1114		57		0,06		0,10	0,06	0,04	0,03		0,06	0,03		1,06	0,09	0,09		0,07	0,04	0,03	0,13		0,04	0,07	0,02	0,08	0,03	0,06	0,04		0,04	0,05
(3*E*)-4,8-Dimethylnona-1,3,7-triene	700	2,04	1119	1116	69	0,01	0,01	0,03	0,02	0,03	0,02	0,03	0,03		0,01	0,01							0,02				0,01	0,03	0,02	0,01				0,01	
2-Ethylhexanoic acid	700	2,16	1119	1123	73	0,04	0,02			0,02	0,02	0,02	0,03		0,02		0,01	0,04	0,03	0,01	0,04	0,01	0,02			0,01	0,01	0,03	0,02	0,01		0,02			0,01
3-Methylundecane	772	1,63	1162	1164	43	0,01	0,03		0,02	0,04	0,08	0,05	0,01	0,05	0,07	0,14	0,03	0,11	0,11	0,03	0,07	0,07	0,05	0,10	0,13	0,11	0,08	0,04	0,13		0,17			0,04	0,09
2,6,6-Trimethylbicyclo[3.1.1]heptan-3-one (3-Pinanone)	772	3,04	1162	1160	83		0,01				0,03	0,02	0,01	0,01	0,03	0,04	0,01	0,02	0,02	0,02	0,01	0,02	0,02	0,05	0,05	0,03	0,03		0,02	0,04	0,08	0,04		0,02	0,04
Benzoic acid	780	3,22	1167	1177	105	0,03	0,01			0,02		0,04	0,03	0,02				0,04	0,06	0,01	0,04		0,01												
1-Phenylpropan-1-one	780	3,64	1167	1176	105	0,13	0,02	0,02	0,05	0,04	0,07	0,05	0,07	0,09		0,05		0,16		0,04	0,30	0,03	0,01		0,06	0,06	0,03			0,06		0,06			0,11
3-Ethyldecane	788	1,63	1171	1170	57	0,01	0,01			0,01	0,04	0,03	0,01	0,02	0,06	0,05	0,77	0,02	0,03	0,03	0,03	0,02	0,04	0,04	0,05	0,04	0,03	0,03	0,04	0,03	0,06	0,03		0,03	0,03
Octanoic acid	788	2,32	1171	1180	60	0,15	0,09	0,05	0,06	0,11	0,08	0,12	0,17	0,12	0,08	0,08	0,04	0,19	0,11	0,07	0,19	0,04	0,11	0,06	0,05	0,03	0,06	0,13	0,05	0,03	0,06	0,12		0,06	0,09
(3*S*,3*as*,7*ar*)-3,6-Dimethyl-2,3,3*a*,4,5,7*a*-hexahydro-1-benzofuran (Anethofuran)	812	2,90	1186	1192	137		0,10			0,01	0,37	0,05	0,01	0,05	0,29	0,10	0,03	0,19	0,20	0,09	0,06		0,17	0,13	0,19	0,14	0,09	0,17	0,17	0,07	0,20	0,16		0,04	0,02
1-(4-Methylphenyl)ethanone	812	3,75	1186	1183	119	0,04	0,03	0,07	0,07	0,04		0,06			0,02		0,01				0,11		0,04					0,08		0,02		0,06			
Dodecane	836	1,67	1200	1200	57	0,16	0,16	0,02	0,16	0,16	0,33	0,26	0,10	0,16	0,27	0,45	0,09	0,31	0,26	0,22	0,23	0,16	0,23	0,36	0,12	0,29	0,31	0,27	0,25	0,17	0,48	0,26		0,13	0,28
Decanal	844	2,42	1205	1206	82	0,02	0,02	0,01	0,01	0,02	0,04	0,04	0,01	0,03	0,03	0,02	0,32	0,03	0,02	0,03		0,01	0,05	0,01	0,01	0,02	0,02	0,03	0,02	0,01		0,02		0,03	0,02
3,5,6-Trimethylnonane	860	1,62	1215	1210	57	0,07	0,07	0,03	0,04	0,03	0,18	0,10	0,03	0,07	0,06	0,18	0,04	0,14	0,12	0,07	0,13	0,07	0,07	0,13	0,06	0,17	0,14	0,09	0,11	0,11	0,18	0,13		0,04	0,12
2,3-Dihydro-1-benzofuran	876	3,60	1225	1224	120	0,02	0,20			0,01	0,03	0,06	0,02				0,01	0,01	0,03		0,04		0,01			0,10						0,02			
[(*E*)-Pent-1-enyl]benzene	884	2,76	1230	1131	117	0,16	0,03	0,07	0,09	0,01		0,01	0,03			0,05									0,08		0,06				0,12				0,08
2-Methyl-5-prop-1-en-2-ylcyclohex-2-en-1-one (Carvone)	908	3,42	1245	1242	82	0,01	0,04				0,18	0,06	0,01	0,02	0,14	0,02		0,05	0,06	0,02	0,03		0,05	0,03	0,08	0,07		0,06	0,05	0,02	0,05	0,04		0,01	0,01
3,3-Dimethylundecane	924	1,68	1255	1275	57	0,02	0,01	0,01	0,02	0,01	0,10	0,04	0,01	0,02	0,09	0,05	0,03	0,04	0,05	0,02	0,01	0,02	0,03	0,05	0,04	0,07	0,05	0,06	0,06	0,03	0,08	0,07		0,06	0,04
1,1-Dibutoxybutane	932	1,94	1260	1229	57	0,55	0,40	0,04			0,43	0,64	0,23							0,61			0,51	0,37	0,41		0,38				0,53	0,48		0,38	0,51
2-(2,6,6-Trimethylcyclohexen-1-yl)acetaldehyde (β-Homocyclocitral)	932	2,98	1260	1254	81		0,03	0,04		0,02	0,02	0,01	0,02	0,10		0,03	0,01		0,02	0,02	0,06	0,02			0,01	0,02	0,03	0,02		0,02	0,06	0,05		0,02	0,05
3-Ethylundecane	940	1,68	1265	1264	57	0,02	0,01	0,01	0,02		0,04	0,04	0,01	0,01	0,04	0,05		0,02		0,01		0,01	0,02	0,02	0,01	0,04	0,02			0,01	0,03	0,02		0,05	
Nonanoic acid	948	2,38	1270	1273	60	0,20	0,04	0,03	0,03	0,05	0,05	0,07	0,12	0,04	0,05	0,03	0,02	0,12	0,06	0,04	0,10	0,02	0,05	0,03	0,04	0,02	0,04	0,07	0,04	0,02	0,03	0,05		0,02	0,04
5-n-Propyldecane	956	1,63	1275		57	0,07	0,03				0,08	0,03	0,03		0,08			0,05	0,03	0,02	0,11	0,03	0,04	0,16		0,19	0,16	0,05	0,03		0,26	0,11		0,10	0,07
2-Methyldodecane	972	1,60	1285	1275	57	0,02		0,11	0,23	0,01	0,22	0,08		0,06	0,25	0,30	0,06	0,03	0,02	0,06		0,01	0,01		0,47	0,01			0,01			0,03		0,18	0,02
Benzene-1,4-diol (Hydroquinone)	980	4,24	1290	1283	110	0,01				1,12		0,08	0,02						0,67				0,03					0,04							
Tridecane	996	1,74	1300	1300	57	0,24	0,09	0,12	0,15	0,11	0,28	0,18	0,13	0,08	0,20	0,19	0,03	0,12	0,08	0,06	0,20	0,01	0,15	0,20	0,06	0,16	0,19	0,22		0,14	0,20	0,12		0,06	0,15
xx-Dimethyldodecane[1]	1028	1,68	1320		57		0,02				0,04	0,03	0,05	0,01		0,03	0,01		0,03			0,02	0,03			0,09	0,03				0,02	0,06		0,02	0,04
4-Ethenyl-2-methoxyphenol	1028	3,78	1320	1316	150	0,03	0,11	0,01	0,02		0,02	0,06	0,03	0,01	0,01	0,01	0,01	0,03		0,01	0,06	0,02	0,02	0,01		0,06		0,03	0,01	0,01	0,03	0,03		0,01	
6-Ethyldodecane	1044	1,65	1330		57	0,06	0,01	0,02	0,14		0,18	0,09		0,02	0,26	0,16	0,05	0,13	0,01	0,07	0,12	0,02	0,01	0,12	0,12	0,15	0,12	0,04		0,05	0,14	0,07	0,04	0,05	0,02
5-Ethyldodecane	1068	1,67	1345		57	0,03	0,01	0,05			0,17	0,07		0,01	0,13	0,11	0,02	0,08	0,01	0,04	0,03			0,05	0,06	0,11	0,03			0,03	0,05	0,05			0,03
4,4,7-Trimethyl-2,3-dihydro-1H-naphthalene (α-Ionene)	1092	3,02	1360		159	0,18	0,22	0,51		0,34	0,33	0,30	0,35	0,18	0,23	0,70	0,20	0,56	0,21	0,29	1,00	0,23	0,27	0,54	0,34	0,56	0,84	0,33	0,24	0,38	1,14	0,87	0,10	0,28	0,71
1,1,6-Trimethyl-2H-naphthalene (Dehydro-ar-ionene)	1092	3,12	1360	1354	157	0,31	0,12	0,21	0,01	0,26	0,19	0,22	0,29	0,10	0,11	0,16	0,06	0,35	0,13	0,12	0,66	0,07	0,14	0,15	0,11	0,13		0,20	0,13	0,12	0,37	0,26		0,07	0,19
Decanoic acid	1100	2,43	1365	1372	60	0,07	0,03	0,02	0,02	0,03	0,02	0,03	0,05	0,03	0,06	0,02	0,01	0,08	0,03	0,02	0,06	0,01	0,02	0,02	0,02	0,01	0,02	0,04	0,03	0,01	0,01	0,03		0,01	0,02
3-Acetyl-4-hydroxy-6-methylpyran-2-one	1100	4,69	1365	1369	43			0,01	0,02	0,08			0,01	0,05	0,02	0,07	0,01	0,08	0,08		0,06	0,03				0,02	0,04		0,06	0,02		0,02			0,01
4-Hydroxybenzaldehyde	1108	4,50	1370	1364	121		0,96						0,01						0,02	0,01	0,01		0,01												
1,2,3-Trimethyl-1H-indene	1116	3,38	1375	1352	143	0,02	0,01	0,01		0,01	0,01	0,01	0,01		0,01	0,01		0,01	0,01	0,01	0,01		0,01			0,01	0,04	0,01		0,01	0,01				0,01
2-Methyltridecane	1124	1,69	1380	1366	57	0,12	0,02	0,05	0,06	0,04	0,09	0,05	0,07	0,04	0,10	0,09	0,04	0,06	0,03	0,03	0,10	0,02	0,03	0,05		0,08	0,07	0,07	0,08	0,03	0,10	0,13		0,05	0,08
Tetradec-1-ene	1140	1,86	1390	1392	57	0,04	0,01			0,01	0,07	0,02	0,07		0,03	0,01	1,26	0,04	0,02	0,02				0,04	0,03	0,05	0,02			0,03	0,02			0,03	0,01
Tetradecane	1156	1,77	1400	1400	57	0,22	0,05	0,10	0,15	0,06	0,16	0,10	0,13	0,05	0,12	0,17	0,05	0,12	0,08	0,07	0,16	0,04	0,09	0,13	0,03	0,12	0,13	0,12	0,07	0,07	0,15	0,10		0,06	0,10
1-(3,6,6-Trimethyl-7,7*a*-dihydro-1H-cyclopenta[c]pyran-1-yl)ethanone	1156	2,88	1382		43	0,06		0,02	0,01				0,02								0,02	0,01		0,02			0,01			0,01					0,02
Vanillin	1156	4,96	1400	1469	151	0,02	0,01				0,02	0,02	0,02	0,02	0,04			0,02					0,02					0,02							
1,7-Dimethylnaphthalene	1164	3,73	1406	1419	156	0,41	0,13	0,26	0,24	0,01	0,15	0,17	0,16		0,02	0,30	0,07	0,21		0,11	0,29	0,12	0,14	0,25	0,18	0,28	0,38	0,13	0,14	0,17	0,45	0,35	0,05	0,12	0,31
Dodecanal	1172	2,44	1410	1386	57	0,04	0,01	0,01	0,01	0,01	0,01	0,01		0,01	0,05	0,01		0,01				0,02	0,01	0,01	0,06		0,01	0,01	0,02	0,03				0,01	0,01

5-Methyltetradecane	1196	1,92	1430		57	0,03			0,01	0,04			0,03		0,01	0,01								0,02	0,01		0,02					0,09			
(5*E*)-6,10-Dimethylundeca-5,9-dien-2-one (Geranylacetone)	1236	2,79	1461	1453	43	0,09	0,04	0,05	0,04	0,04	0,06	0,05	0,06	0,03	0,04	0,04	0,01	0,04	0,04	0,03	0,08	0,02	0,06	0,03	0,06	0,04	0,04	0,06	0,04	0,02	0,04	0,05		0,03	0,04
2,6,10-Trimethyltridecane	1252	1,68	1473		57	0,19	0,16	0,15	0,12	0,11	0,27	0,15	0,13	0,53	0,21	0,15	0,06	0,16	0,10	0,12	0,32	0,10	0,11	0,16	0,11	0,22	0,20	0,22	0,01	0,14	0,01	0,27		0,09	0,16
(5*Z*)-2,6,10-Trimethylundeca-1,5,9-triene	1260	2,10	1479		69	0,06	0,09		0,02	0,07		0,06	0,06	0,03	0,05					0,01	0,07		0,09			0,02	0,09	0,17		0,04		0,06		0,05	0,06
(*E*)-4-(2,6,6-Trimethylcyclohexen-1-yl)-but-3-en-2-one (trans-β-Ionone)	1284	2,89	1497	1486	177	0,04	0,01	0,03		0,02	0,05	0,05		0,11	0,05	0,04	0,05	0,28	0,28	0,17		0,03		0,06	0,13	0,06	0,03	0,06	0,06	0,06	0,12	0,18		0,03	0,15
Pentadecane	1300	1,69	1509	1500	57	0,45	0,09	0,09	0,12	0,18	0,15	0,17	0,28	0,06	0,29	0,14	0,05	0,20	0,10	0,08	0,18	0,09	0,13	0,03	0,03	0,12	0,04	0,18	0,09	0,14	0,17	0,23		0,09	0,19
Tridecan-2-one	1300	2,22	1509	1496	58	0,02	0,03	0,03	0,02		0,01	0,03	0,03	0,02	0,01	0,02			0,01	0,01	0,04		0,01	0,01	0,03		0,02	0,02	0,02	0,02				0,02	0,03
Dibenzofuran	1324	3,75	1527		168		0,01	0,01	0,01	0,02	0,02	0,02	0,02	0,01	0,01	0,02		0,02	0,01	0,01	0,03	0,01	0,02	0,01	0,02	0,01	0,02	0,02	0,01	0,01	0,02	0,02		0,01	0,02
5-Hydroxy-3-methyl-2,3-dihydroinden-1-one	1332	3,70	1533		162	0,05		0,06	0,05	0,06	0,04	0,07	0,06	0,01		0,04						0,02	0,06	0,03		0,01		0,06	0,03	0,02	0,04	0,03	0,01	0,02	0,03
1-(4-Hydroxy-3-methoxyphenyl)ethane-1,2-diol (Vanylglycol)	1340	3,05	1539	1522	121				0,01		0,03	0,09		0,01	0,41	0,02	0,01			0,02	0,11		0,01		0,48	0,28	0,01	0,01						0,01	
4,4,7*a*-Trimethyl-6,7-dihydro-5*H*-1-benzofuran-2-one (Dihydroactinidiolide)	1348	3,82	1546	1532	111	0,14	0,05	0,14	0,14	0,07	0,09	0,11	0,19	0,15	0,08	0,08	0,05	0,11	0,28	0,15	0,55	0,04	0,09	0,07	0,06	0,06	0,05	0,11	0,09	0,07	0,13	0,19	0,02	0,05	0,16
2,4-Dimethyl-3-ethyldodecane,	1356	1,51	1552		57		0,03	0,04	0,24	0,01	0,05	0,06	0,03	0,04	0,67	0,19	0,04	0,11	0,02	0,01	0,22	0,01	0,02	0,04	0,15	0,73	0,12	0,03	0,01	0,04	0,06			0,06	
2-Methylhexadecane	1364	1,55	1558		57	0,20	0,01			0,01		0,10	0,07	0,04					0,02	0,02	0,01	0,07	0,02	0,07				0,03	0,01	0,05		0,14			0,07
Dodecanoic acid	1380	2,02	1570	1567	60	0,40	0,25	0,39	0,45	0,35	0,27	0,31	0,22	0,10	0,15	0,37	0,03	0,56	0,07	0,10	0,19	0,13	0,26	0,24	0,30	0,14	0,37	0,34	0,24	0,15	0,25	0,21	1,47	0,17	0,25
3-Hydroxy-4-methoxybenzoic acid (Isovanillic acid)	1388	3,42	1576	1560	168	0,01							0,01	0,01				0,01	0,01	0,01	0,02														
Hexadecane	1420	1,52	1600	1600	57	0,51	0,08	0,10	0,11	0,09	1,13	0,09	0,30	0,56	0,14	0,11	0,06	0,12	0,04	0,04	0,28	0,03	0,02	0,11	0,19	0,46	0,24	0,08	0,04	0,07	0,09	0,11		0,04	0,09
2,6,10-Trimethylpentadecane	1468	1,44	1657	1633	57		0,07	0,16	0,10		0,17	0,08	0,32	0,21	0,10		0,08	0,11	0,06	0,09		0,09	0,08	0,11	0,69		0,17	0,09	0,09				0,02	0,06	0,16
4-Hydroxy-4-(2,6,6-trimethylcyclohexen-1-yl)-but-3-en-2-one (4-Hydroxy-β-ionone)	1468	2,65	1658		43				0,03			0,05					0,03		0,07	0,09											0,07				
Tridecanoic acid	1476	1,87	1667	1664	73	0,02		0,01	0,01	0,01	0,01	0,01	0,01	0,01	0,04	0,01		0,02				0,01	0,01	0,01	0,02		0,01	0,01	0,01		0,01		0,02	0,01	0,01
3-Hydroxy-5,6-epoxy-4-(2,6,6-trimethylcyclohexen-1-yl)-but-3-en-2-one (3-Hydroxy-5,6-epoxy-β-ionone)	1500	2,54	1695	1674	123		0,01						0,04				0,03		0,06																
Heptadecane	1508	1,48	1705	1700	57	1,20	0,06	0,09	0,03	0,19	0,07	0,08	0,56	0,02		0,11	0,08	0,22	0,05	0,05	0,64	0,27	0,08	0,11	0,18	0,65	0,19	0,11	0,07	0,06	0,04	0,06		0,04	0,09
Pentadecan-2-one	1508	1,82	1705	1698	58	0,05	0,03	0,04	0,04	0,02	0,02	0,02	0,03	0,01	0,01	0,02						0,04	0,02	0,04	0,04		0,02	0,02	0,01	0,10			0,03	0,02	0,02
Pentadecanal	1524	1,81	1724	1702	43	0,05	0,02	0,03	0,01	0,01	0,02	0,02	0,02	0,02	0,01	0,01		0,09	0,01		0,01	0,02	0,01	0,02	0,01	0,02	0,02	0,01	0,01	0,01	0,02	0,09	0,03	0,01	
(*Z*)-3,7,11,15-Tetramethylhexadec-2-ene (*cis*-Phytene)	1532	1,47	1733	1830	57		1,02	0,17	0,11	0,28	0,25	0,08		0,12		0,05	0,04	0,56	0,05	0,06	0,28		0,12	0,08			0,10	0,18	0,10	0,11	0,18	0,31		0,04	
5-Methylheptadecane	1548	1,56	1752		57	0,42	0,02	0,02	0,10	0,08	0,44		0,23		0,03	0,09	0,05	0,12				0,13			0,02					0,04					
Tetradecanoic acid	1556	1,82	1762	1768	73	0,62	0,32	0,54	0,60	0,61	0,37	0,45	0,35	0,23	0,17	0,46	0,07	1,10	0,13	0,15	0,27	0,23	0,37	0,40	0,51	0,18	0,46	0,41	0,33	0,31	0,30	0,41	1,58	0,26	0,35
4-Methylheptadecane	1564	1,41	1771		57	0,06		0,16			0,04		0,07	0,13			0,03	0,05	0,01	0,01	0,08			0,15	0,02	0,22	0,12	0,04		0,11				0,03	
Octyl benzoate	1564	2,08	1771	1792	105	0,11	0,03	0,05	0,06	0,05	0,04	0,06	0,06	0,04	0,03	0,07		0,06	0,03	0,02	0,06	0,06	0,04	0,08	0,12		0,05	0,02	0,04	0,04	0,05	0,09	0,02		0,07
(*E*)-3,7,11,15-Tetramethylhexadec-2-ene (*trans*-Phytene)	1580	1,43	1790	1830	70		0,10	0,20	0,16	0,23		0,15			0,17	0,14			0,04	0,06	0,35		0,13	0,13			0,14	0,08	0,12					0,08	0,18
(6*S*,7*ar*)-6-Hydroxy-4,4,7*a*-trimethyl-6,7-dihydro-5*H*-1-benzofuran-2-one (Loliolide)	1580	3,45	1790	1793	43		0,01	0,40	0,41		0,04	0,07	0,22			0,03	0,02	0,06	0,03	0,05		0,02	0,07	0,07	0,04		0,02	0,07		0,06	0,11	0,13		0,02	0,04
6-Methoxy-3*H*-1,3-benzoxazol-2-one	1580	3,69	1790		165						0,02			0,06			0,42	2,67	1,09										0,30						
Octadecane	1588	1,45	1800	1800	57	1,19	0,06				0,50		0,70	0,11	0,22	0,05	0,18	0,22	0,04	0,04	0,04	0,04		0,05	0,68	1,63	0,15	0,09		0,04	0,13	0,13	0,02	0,03	
Hexadecanal	1604	1,75	1824	1817	57		0,10	0,03	0,04	0,06	0,03	0,07	0,03		0,02		0,01	0,01	0,02	0,01	0,02	0,03	0,04	0,03		0,05	0,03	0,02	0,01	0,04			0,03	0,02	0,02
(*E*)-3,7,11,15-Tetramethylhexadec-2-ene (Phytene-2)	1612	1,46	1835	1830	70	0,33	0,11		2,05	1,39	0,50		1,23	0,18				0,57	0,21		2,16		0,32					0,44					1,14		
7,11,15-Trimethyl-3-Methylidenehexadec-1-ene (Neophytadiene)	1620	1,51	1847	1838	68	3,74	1,68	4,24	4,41	2,56	2,72	2,78	1,17	2,08	1,61	4,14	1,28	3,85	1,87	1,88	4,33	2,05	2,07	4,13	3,57	3,95	5,36	2,44	2,22	2,84	7,96	8,09	6,36	2,56	5,38
(3*Z*)-3,7,11,15-Tetramethylhexadec-1,3-diene (Neophytadiene, isomer 1)	1628	1,46	1859	1838	68			0,03		2,43		0,34	0,04			0,03			0,02				0,08			2,11	0,13	2,61			0,06	0,06	0,19		0,12
Pentadecanoic acid	1628	1,80	1859	1867	73	0,12	0,03	0,06	0,06	0,06	0,03	0,12	0,07	0,05	0,05	0,02	0,01	0,12	0,02	0,04	0,08	0,02	0,05	0,03	0,09		0,08	0,07	0,03	0,02	0,05	0,18	0,13	0,02	0,03
Nonyl benzoate	1636	2,07	1871	1847[2]	105	0,23	0,11	0,15	0,11	0,19	0,16	0,24	0,24	0,19	0,12	0,12	0,03	0,29	0,13	0,09	0,26		0,24	0,09			0,10	0,20	0,16		0,12		0,24	0,05	0,11
(3*E*)-3,7,11,15-Tetramethylhexadeca-1,3-diene (Neophytadiene, isomer 2)	1644	1,54	1882	1838	81		1,65	3,42	3,29	0,01	2,34	2,53	2,12	2,13	1,53	2,98	0,83	3,39	1,56	1,45	1,41	1,33	0,09	2,90	2,52	2,71	4,72		2,34	2,02	5,98	5,38	4,06	1,82	4,67
(*E*)-3-(4-Hydroxy-3-methoxyphenyl)prop-2-enoic acid (Ferulic acid)	1644	3,06	1882	1897	194	0,06		0,04				0,03	0,02	0,01				0,02	0,01				0,01					0,01							
Hexadecan-1-ol	1652	1,40	1894	1866	57							0,03												0,15							0,05	0,11	0,11		0,13
Nonadecane	1660	1,44	1906	1900	57	1,93	0,21	0,14	0,06	0,10	0,09	0,09	0,93	0,13	0,15	0,09	0,40	0,33	0,06	0,06	0,54	0,05	0,04		0,21		0,09	0,05	0,08	0,07	0,07			0,10	0,07
Heptadecan-2-one	1660	1,73	1906	1904	58	0,25	0,16	0,30	0,31	0,14		0,12	0,08									0,31	0,04	0,26	0,31	0,16	0,07	0,05		0,29			0,49	0,07	0,07
Isoalkane[1]	1676	1,35	1929		57	0,42		0,04	0,04		0,18	0,02	0,39	0,04	0,19	0,04	0,09	0,09	0,03		0,11	0,02	0,02	0,04	0,41	0,38	0,10	0,04	0,03	0,05	0,05	0,02		0,04	0,05
3-Methyl-2-(3,7,11-trimethyldodecyl)furan	1676	1,61	1929		95	0,18			0,04	0,06	0,08	0,05	0,11	0,04	0,11	0,02	0,03		0,02	0,01	0,13	0,02	0,04	0,03	0,22	0,18	0,05	0,05		0,02		0,07			0,04
Methyl hexadecanoate	1676	1,69	1929	1926	74	0,33						0,06	0,17		0,07		0,02	0,09			0,25			0,06	0,09	0,07				0,07		0,10		0,01	
(5*E*,9*E*)-6,10,14-Trimethylpentadeca-5,9,13-trien-2-one (Farnezyl acetone)	1676	1,92	1929	1918	43	0,03	0,01			0,04	0,03	0,02	0,07	0,03	0,03	0,02		0,04	0,02	0,03	0,10		0,03		0,02		0,02			0,02	0,02	0,03	0,54	0,01	
2,3,4- Trimethylheptadecane	1684	1,42	1941		57		0,09	0,05	0,03	0,05		0,04	0,02	0,02	0,04	0,03	0,09		0,01	0,03			0,03	0,05	0,04					0,06	0,02			0,04	
(*Z*)-Hexadec-9-enoic acid (Palmitoleic acid)	1684	1,86	1941	1961	55									0,01					0,09	0,05	0,19		0,05						0,04						
3,7,11,15-Tetramethylhexadec-1-en-3-ol (Isophytol)	1692	1,58	1953		71		0,05		0,02								0,10				0,45		0,04											0,03	
2,6,10,15-Tetramethylhexadecane	1700	1,37	1965		57	0,38	0,04		0,08		0,43	0,04			0,34	0,09		0,39			0,08	0,04		0,06	0,21		0,18	0,09	0,03	0,03	0,06			0,03	
Hexadecanoic acid	1700	1,77	1965	1968	73	3,08	1,61	1,67	1,91	5,33	1,53	3,63	0,96	6,19	0,92	2,14	0,72	7,80	2,35	3,42	2,53	0,86	1,34	1,95	2,06	1,24	4,14	4,79	12,19	1,27	2,08	2,98	6,55	2,48	3,86
Ethyl hexadecanoate	1716	1,68	1978	1993	88	0,79	0,11	0,13					0,59	0,21			0,14					0,16								0,08					0,17
(6*E*,10*E*)-3,7,11,15-Tetramethylhexadeca-1,6,10,14-tetraen-3-ol (Geranyllinalool)	1716	1,82	2000	2034	69		0,05	0,04	0,04	0,13		0,03		0,04					0,05	0,01		0,02		0,03			0,04	0,03			0,03	0,04			
Eicosane	1724	1,44	2000	2000	57	1,31				0,16	0,30	0,03	0,08	0,14	0,06		0,66	0,47	0,06	0,02	0,55	0,20	0,03					0,03	0,06		0,02	0,02	0,03		0,07
Methyl (9*Z*,12*Z*,15*Z*)-octadeca-9,12,15-trienoate (Methyl linolenate)	1724	1,94	2000		69	0,18		0,02	0,02	0,03		0,03	0,09								0,31		0,03							0,03	0,09	0,15			
Octadecanal	1732	1,74	2014	1995	57	0,11	0,02	0,02		0,04	0,02	0,04	0,04	0,06	0,03			0,22		0,02	0,05	0,02		0,07		0,06		0,02		0,01		0,01		0,01	0,01
N-Ethyltetradecanamide	1747	2,04	2043	2067[2]	87	1,16							0,93				0,07	0,14			0,17			0,01				0,01			0,02	0,01			
Heptadecanoic acid	1756	1,55	2057	2071	73	0,08	0,07	0,10	0,03	0,22					0,01					0,02	0,05			0,09	0,07		0,05	0,02	0,02	0,02	0,03	0,09			0,04
6-Methyl, 7-n-propylheptadecane	1764	1,42	2071		57	0,83	0,19	0,01	0,01		0,10		0,17	0,04			0,73	0,19			0,10				0,06	0,95				0,05	0,02	0,05		0,02	0,01
Heneicosane	1780	1,46	2100	2100	57	0,94	0,25	0,09	0,12	0,20	0,41		0,93	0,14	0,58	0,17	0,72	0,08	0,12		0,65	0,06		0,01	0,12	0,94	0,06	0,04	0,04	0,11		0,07			
(*E*,7*R*,11*R*)-3,7,11,15-Tetramethylhexadec-2-en-1-ol (Phytol)	1796	1,69	2129	2114	71	7,77		2,67	3,19	2,38	1,93	3,09	4,43			3,27	1,02	2,68	1,55	1,84	0,05	2,37	4,60	5,50	3,34	1,99	3,79	4,85		1,92	2,73	5,97			4,39
(9*Z*,12*Z*)-Octadeca-9,12-dienoic acid (Linoleic acid)	1796	1,96	2129	2133	67	4,77	0,13	9,49	0,33	0,56	0,08		2,47	0,92	0,10	0,04	0,80		1,49		2,89	0,17	4,34	5,56	0,11	1,07	7,48	8,73		0,39	0,72	0,98	1,52	0,23	11,44
2-Methyl-3-ethyl-nonadecane	1804	1,44	2143		57	0,90		0,45	0,05		0,51		0,09		0,07		0,28							0,42	0,07	0,33	0,54			0,10					0,43
(*Z*)-Octadec-9-enoic acid (Oleic acid)	1804	1,88	2143	2164	55	0,96	0,81	0,02	0,27		2,17	0,07	0,07	23,18	0,38	0,20	0,17	1,22	2,80	0,68	0,78	0,07	0,31		1,00	0,50			0,71	0,25	0,10	0,73	0,14	0,08	0,16
(9*Z*,12*Z*,15*Z*)-Octadeca-9,12,15-trienoic acid (Linolenic acid)	1804	2,02	2143	2139	79	1,26	5,61		8,37	8,46	2,87	0,22	0,58	9,28	1,52	8,72	1,50	7,64	6,35	4,59	11,22	4,22		0,38	3,67	3,19		1,11	2,36	2,85	17,90	13,68	25,52	4,19	
Octadecanoic acid	1812	1,80	2157	2172	43		0,49		0,24	2,19	0,54	0,74	0,19	0,40	0,15	0,06	0,19	6,84	0,51	1,19			0,32	0,14	0,21	0,18	0,16	0,29	0,22		0,23	0,87			0,06
2,6-Dimethoxy-4-prop-2-enylphenol (4-Allylsyringol)	1812	2,36	2157		91	0,12		0,17	0,14	0,01		0,02					0,04	0,13		0,05	0,01	0,07		0,11	0,06	0,13	0,08	0,04	0,05	0,11	0,39	1,20	0,68	0,06	0,07
3-Methylheneicosane	1820	1,45	2171		57	0,66	0,21	0,02					0,14	0,06	0,24		0,84				0,35				0,15	0,11				0,04		0,06			
Ethyl (9*Z*,12*Z*)-octadeca-9,12-dienoate (Ethyl linoleate)	1820	1,89	2171		79	2,58							0,92				1,07	3,21		6,33	14,21														
Docosane	1836	1,47	2200	2200	57	1,04		0,07	0,14	0,21	1,12	0,04	1,63	0,17	1,53	0,20	2,03		0,13	0,03	1,04	0,23	0,04	0,06	0,19	1,98	0,07	0,04	0,11	0,11	0,09	0,07	0,04	0,19	0,04
Ethyl octadecanoate	1836	1,68	2176	2195	88				0,01			0,01	0,38		0,01						0,22												0,01		
Nonadecane-2,4-dione	1836	1,79	2184	2200	100							0,07										0,07			0,05					0,05					0,02

[(*E*,7*R*,11*R*)-3,7,11,15-Tetramethylhexadec-2-enyl] acetate (Phytyl acetate)	1844	1,70	2217		43	0,72	1,91	0,12	0,21			0,34	0,34	1,41	0,28	0,22	0,21	1,76	27,96		0,45	0,31	0,40	0,33	0,34	0,27	0,23	0,53	15,72	0,22	0,08	0,49		1,37	0,40
11-Methyldocosane	1852	1,43	2233		57	0,44	0,12	0,04				0,02	0,08		0,27	0,02	2,30	0,48		0,02		0,09		0,02	0,22					0,08	0,09		0,09		0,03
2-Methyldocosane	1868	1,39	2267		57				0,05					0,02	0,43	0,02								0,04	0,35	1,50	0,09			0,04				0,03	
N-Ethylhexadecanamide	1868	1,90	2267[3]	2267	87	8,84			0,01		0,05		9,04	0,05	0,25		1,28	0,82		0,03	5,83			0,04	0,12	0,25		0,15			0,14	0,03			
Tricosane	1884	1,41	2300	2300	57	2,57	0,32	0,26	0,24	0,46	2,16	0,26	3,18	0,37	2,12	0,26	6,74	0,94	0,20	0,12	1,54	0,29	0,18	0,17	0,54	2,60	0,17	0,14	0,28	0,26	0,51	0,33	0,20	0,12	0,18
Oxiran-2-ylmethyl hexadecanoate (Glycidyl palmitate)	1884	1,80	2300	2294	57		0,02	0,04	0,04	0,11	0,10	0,05		0,08	0,08	0,04		0,16	0,05	0,02		0,02	0,02	0,02			0,05	0,06	0,01	0,03	0,07	0,05		0,03	0,05
Henicosanal	1900	1,59	2333	2332	57	0,13	0,03	0,04				0,06		0,04	0,03			0,03		0,01			0,06	0,07			0,04	0,07		0,07					
6-Methyltricosane	1908	1,34	2350		57		0,03	0,05	0,11			0,11			0,19	0,10	0,12	0,22					0,13	0,09	0,32		0,12				0,19			0,04	
Eicosanoic acid	1916	1,60	2367	2380	43			0,03	0,02	0,04		0,03			0,06		0,78							0,03	1,42		0,02			0,03		0,01		0,02	
5-Methyl-5-(4,8,12-trimethyltridecyl)oxolan-2-one (4,8,12,16-Tetramethylheptadecan-4-olide)	1916	1,75	2367	2364	99	0,02		0,11	0,05	0,04	0,03	0,02		0,03		0,06			0,02	0,02		0,09	0,03	0,04	0,10	0,03	0,06	0,05	0,03	0,03				0,06	0,05
N-Ethylheptadecanamide	1916	1,76	2367	2367[2]	87	0,15							0,16				0,01	0,02			0,03														
(*E*,7*R*,11*R*)-3,7,11,15-Tetramethylhexadec-2-enyl butyrate (Phytyl butyrate)	1924	1,51	2383		85																			0,08		0,07								0,52	
(6*E*,10*E*,14*E*,18*E*)-2,6,10,14,18-Pentamethylicosa-2,6,10,14,18-pentaene	1924	1,56	2383		69	0,11	0,12		0,19	0,13		0,08		0,02	0,15						0,20		0,08					0,12		0,08	0,16				
Tetracosane	1932	1,35	2400	2400	57	0,59	0,18			0,17	2,41		1,89	0,09	9,79		4,28	0,24	0,07	0,02	0,08	0,30	0,09		0,85	3,24		0,08		0,21					
Docosanal	1940	1,60	2422	2430	57	0,31	0,06	0,04	0,03	0,04		0,04		0,06		0,06		0,16	0,01			0,03	0,13	0,03		0,02	0,05	0,07	0,02	0,08	0,03	0,16	0,09	0,25	0,04
9-Methyltetracosane	1948	1,38	2444		57	1,84		0,03			0,13				0,05					0,03							0,04				0,25	0,20	0,29	0,06	0,09
(7*Z*,10*Z*,13*Z*)-Hexadeca-7,10,13-trienal	1948	1,96	2444			0,14	0,15	0,05	0,09	0,04		0,03	0,06		0,07	0,08			0,08	0,01	0,22	0,05	0,02	0,07	0,13	0,12	0,11			0,10	0,14	0,14	0,40	0,01	0,09
3,4,13-Trimethyltricosane	1956	1,39	2467		57		0,06	0,05	0,04	0,12	0,16	0,07	0,07	0,03	0,16	0,04							0,02	0,04	0,14	1,22	0,06		0,02	0,14				0,06	
(2*Z*,6*E*,10*E*)-3,7,11,15-Tetramethylhexadeca-2,6,10,14-tetraen-1-ol	1956	1,78	2467		69		0,02	0,04		0,10				0,07									0,04												0,03
N-Ethyloctadecanamide	1956	1,89	2467[3]	2467	87	6,23					0,07		35,22	0,07	0,30		1,71	1,12	0,01	0,03	18,13			0,02	0,09	0,29		0,27			0,06	0,02			
Pentacosane	1964	1,47	2489	2500	57	0,37	0,53	0,62	0,40	0,53	3,32	0,73	3,01	0,60	3,07	0,49	7,89	0,89	0,27	0,45	2,71	0,53	0,41	0,51	3,84	4,24	0,49	0,26	0,33	0,57	0,75	0,87	0,52	0,67	0,45
Methyl (5*Z*,11*Z*,14*Z*,17*Z*)-icosa-5,11,14,17-tetraenoate	1964	2,08	2489		79		0,02	0,30	0,20	0,13	0,19	0,12				0,17		0,20	0,01	0,06		0,08	0,08	0,10	0,13		0,06	0,15	0,05	0,03	0,30	0,40		0,08	0,28
2,5-Dimethyltetracosane	1972	1,63	2511		57	0,04	0,04					0,04						0,12				0,13	0,02	0,02	0,07	0,40	0,02							0,08	
(*Z*)-Octadec-13-enal	1972	1,78	2511		69		0,04	0,10	0,10		0,16	0,07		0,09	0,13			0,12	0,02										0,04		0,13	0,11	0,48	0,10	0,11
Tricosanal	1980	1,75	2533		57	0,28				0,74						0,09		0,12		0,04		0,06	0,15	0,09	0,07	0,33				0,08				0,10	
5-Methylpentacosane	1988	1,46	2556		57	0,89					0,05		0,07	0,08			2,28				0,01	0,12		0,03		1,11									
2-Methylpentacosane	1996	1,55	2558		57				0,04		0,38		0,02	0,04	0,35	0,03					0,18	0,30	0,02	0,04	0,65									0,05	
(*E*,7*R*,11*R*)-3,7,11,15-Tetramethylhexadec-2-enyl hexanoate (Phytyl hexanoate)	1996	1,79	2577		68				0,01			0,03		0,04		0,01						0,02		0,01					0,05					0,04	
Docosanoic acid	1996	1,87	2578	2568	43	0,23		0,05				0,01	0,07						0,03		0,09	0,04		0,08		0,22	0,01	0,03	0,03	0,07		0,06		0,07	
Hexacosane	2004	1,60	2600	2600	57	0,61	0,25	0,25	0,02	0,17	3,14	0,13	1,82		2,64	0,19	5,39	1,02	0,07	0,16	0,52	0,12	0,17	0,15	0,72	3,09	0,28	0,12	0,12	0,10	0,35	0,18	0,18	0,10	0,18
Hexacos-1-ene	2004	1,63	2600	2595	97	0,37	0,09			0,05	0,03			0,04		0,05	2,13	0,09	0,04	0,03	0,16	0,03		0,03	0,23		0,07	0,02	0,02	0,03	0,09	0,05		0,06	0,02
Tetracosanal	2020	1,95	2633	2632	57	0,08	0,05	0,04	0,06	0,05		0,08		0,05	0,05	0,07	0,39	0,02	0,01		0,06	0,05	0,09	0,08	0,17	0,15	0,05	0,05	0,06	0,11	0,04	0,07	0,07	0,12	0,04
4-Methylhexacosane	2028	1,59	2650		57	0,48	0,07	0,04	0,04			0,03		0,20		0,02	2,07		0,02			0,24	0,02	0,07	0,10		0,05		0,02	0,04	0,06	0,03		0,06	0,03
2-Methylhexacosane	2036	1,66	2667		57		0,07	0,03	0,03			0,16	0,10	0,03	0,37	0,03	0,79	0,08	0,02	0,03		0,06	0,02	0,03	0,11	0,81	0,04			0,03	0,05	0,04		0,06	0,02
N-Ethylicosanamide	2036	2,37	2667	2673	87	1,37							0,57				0,47	0,17			0,08							0,01							
2,3-Dihydroxypropyl (9*Z*,12*Z*,15*Z*)-octadeca-9,12,15-trienoate (1-mono-linolenin)	2044	2,42	2683	2702	117		0,10	0,30	0,41	0,07		0,02											0,08	0,42			0,58	0,16		0,09	0,70	0,65			
Heptacosane	2052	1,75	2700	2700	57		2,22	1,84	1,87	1,66	4,20	1,49	0,45	1,51	3,12	1,81	5,44	1,05	0,84	0,86	1,42	0,97	1,50	1,55	0,87	2,67	1,32	1,06	1,10	1,06	2,26	1,58	1,44		1,47
tetracosan-1-ol	2060	2,09	2717	2698	57			0,07	0,36		0,22	0,41	0,35		0,08	0,48		0,45		0,09		0,28		0,22	0,10	0,73	0,43	0,29		0,62	0,10	0,22		0,25	0,21
11-Methylheptacosane	2068	1,81	2733		57	0,17	0,09	0,06	0,05	0,05	0,05	0,07	0,02	0,04		0,06	0,07		0,02	0,02	0,21	0,05	0,06	0,05	0,12	0,08	0,06	0,02	0,03		0,04	0,04	0,03	0,07	0,03
Pentacosanal	2068	2,20	2733	2738	57		0,08	0,05	0,09	0,04	0,06	0,10	0,12	0,05	0,05	0,03	1,08	0,06	0,03	0,04	0,21	0,03	0,03	0,07	0,05		0,04	0,05	0,02	0,06	0,06	0,07	0,06	0,08	0,07
(*E*,7*R*,11*R*)-3,7,11,15-Tetramethylhexadec-2-enyl octanoate (Phytyl octanoate)	2084	2,22	2766		57	0,21	0,13	0,15	0,20	0,14	0,15	0,34	0,37	0,18	0,13	0,17	0,16	0,33	0,13	0,04	0,26	0,06	0,22	0,17	0,24	0,14	0,11	0,18	0,20		0,22	0,59	0,06	0,40	0,13
2-Methylheptacosane	2084	2,23	2762	2761	57				0,04		0,02	0,02	0,01	0,02	0,34		0,54				0,02		0,02			0,01					0,02				
N-Ethylhenicosanamide	2084	2,73	2767	2767[2]	87	0,04											0,01																		
(*Z*)-Docos-13-enamide (Erucamide)	2092	3,20	2783	2793	59	0,04	0,01	0,07	0,04	0,08	0,04	0,24	0,02	0,02	0,01				0,07	0,07	0,04		0,01	0,06	0,09	0,04	0,04		0,04	0,01	0,04	0,06	0,05		0,16
Octacosane	2100	1,96	2800	2800	57	0,19	0,50	0,23	0,20	0,69	2,45	0,33	0,18	0,22	1,85	0,19	3,55	0,22	0,40	0,16	0,21	0,13	0,20	0,21	0,41	1,79	0,33	0,16	0,12	0,12	0,31	0,18	0,40	0,12	0,14
Tetracosyl acetate	2108	2,36	2813	2808	43		0,02	0,06		0,04		0,06										0,06											0,04		
Hexacosanal	2124	2,54	2838	2832	57		0,17	0,18	0,21	0,19	0,25	0,40	0,01	0,31	0,28	0,21	0,07	0,17	0,10	0,30	0,06	0,26	0,20	0,29	0,35	0,20	0,18		0,14	0,02	0,08	0,08	0,24	0,44	0,12
(6*E*,10*E*,14*E*,18*E*)-2,6,10,15,19,23-Hexamethyltetracosa-2,6,10,14,18,22-hexaene (Squalene)	2124	2,62	2838	2826	69	2,32	0,62	1,73	0,05	0,73	0,05	2,30	0,06	0,11	0,63	0,26							1,48	0,59		0,40	1,53	1,77		0,01	0,05		1,79		
(6*E*,10*E*,14*E*,18*E*)-2,6,10,14,18,22- Hexamethyleetracosa-2,6,10,15,19,23-hexaene (Supraene)	2124	2,67	2838	2826	69	0,42	0,05	0,03	0,04	0,04	0,03	0,02	0,77			0,02				0,01			0,06	0,21	0,03		0,04	0,05	0,05	0,01	0,07		0,25	0,02	0,02
11,15-Dimethylheptacosane	2132	2,06	2850	2863	57	0,14	0,02	0,05	0,03	0,02	0,18	0,05	0,26	0,10	0,18	0,02	0,28	0,03	0,02	0,02	0,08	0,01	0,05	0,02	0,08	0,58	0,05			0,05	0,03	0,02			0,02
(2*S*,5*R*,9*S*)-9-Acetyl-9-hydroxy-2,7,8-trimethyl-2-[(4*R*,8*R*)-4,8,12-trimethyltridecyl]-1-oxaspiro[4.4]non-7-en-6-one (α-Tocospiro A)	2140	3,07	2863	2860	43	0,33		0,03				0,02	0,28	0,02		0,04		0,03			0,08	0,02		0,02				0,03		0,03	0,13	0,08			0,02
N-Ethyldocosanamide	2140	3,20	2863	2867[2]	87	1,24							0,17				0,17	0,12			0,05														
Nonacosane	2156	2,26	2888	2900	57	0,06	7,92	6,46	5,24	5,11	7,65	4,17	0,18	2,73	4,30	5,44	2,22	0,80	0,52	0,82	0,59	3,51	5,86	4,95	1,56	3,66	2,57	3,57	3,31	5,88	4,25	1,69	2,86	7,91	0,01
(2S,5R,9S)-9-Acetyl-9-hydroxy-7,8-dimethyl-2-[(4R,8R)-4,8,12-trimethyltridecyl]-1-oxaspiro[4.4]non-7-en-6-oneα-Tocospiro B	2156	3,16	2888	2882	43	0,12	0,01	0,02				0,02		0,01				0,03	0,01	0,01	0,07	0,01	0,01	0,02					0,01	0,04	0,03	0,12			
Hexacosan-1-ol	2164	2,78	2900		57			1,32	0,59		1,48	3,04	0,21	0,03		1,15	1,62	1,50	0,94	1,49		1,72		0,78	1,09	1,17	0,75	0,49		4,34	0,32	0,45	0,03	3,97	0,90
7,21-Dimethyloctacosane	2172	2,16	2913		57	0,09	0,08	0,06	0,06	0,03			0,24	0,03		0,03	0,19				0,04	0,04	0,02	0,04	0,03	0,20	0,03			0,05		0,04		0,45	0,04
Heptacosanal	2180	2,95	2925		57	0,06		0,10	0,10	0,07	0,10	0,06			0,05	0,12	0,05	0,04	0,03	0,01	0,01	0,13	0,10	0,12	0,18	0,07	0,09	0,08	0,02	0,10	0,09	0,06	0,09	0,03	
(*E*,7*R*,11*R*)-3,7,11,15-Tetramethylhexadec-2-enyl decanoate (Phytyl decanoate)	2204	2,94	2963		57		0,04		0,05	0,03		0,07			0,07	0,03	0,04		0,04	0,02			0,05	0,03		0,02	0,03				0,03		0,02		0,02
Triacontane	2220	2,62	2988	3000	57	0,01	0,20	0,14	0,14	0,09	1,61	0,06	0,11	0,06	1,20	0,13	1,81	0,03	0,02	0,10	0,01	0,10	0,09	0,19	0,27	0,57	0,22	0,11	0,05	0,16	0,10	0,01	0,08	0,15	0,11
β-Sitosterol –H_2O[1]	2228	3,94	3004		81		0,04		0,02	0,09		0,05		0,05				0,03	0,04		0,05														
Octacosanal	2260	3,51	3040	3036	57		2,16	2,11	1,90	2,04	1,80	2,83	0,01	3,79	2,59	2,47	0,99	1,73	2,70	2,24	0,32	1,49	2,39	2,45	2,66	1,22	1,76	1,44	2,37	2,22	3,56	1,98		2,62	2,17
(8*S*,9*S*,10*R*,13*R*,14*S*,17*R*)-4,4,10,13-Tetramethyl-17-[(2*R*,3*E*)-6-methylhepta-3,5-dien-2-yl]-1,2,3,5,8,9,11,12,14, 15,16,17-dodecahydrocyclopenta[a]phenanthrene (4,4-Dimethyl-cholesta-6,22,24-triene)	2284	4,82	3066		81	0,56	0,09	0,33		0,08		0,05	0,18											0,29	0,08		0,18			0,02					
(2*R*)-2,7,8-Trimethyl-2-[(4*R*,8*R*)-4,8,12-trimethyltridecyl]-3,4-dihydrochromen-6-ol (γ-Tocopherol)	2284	5,01	3066	3057	151	0,06			0,24	0,08		0,07	0,04		0,01	0,27			0,04		0,01	0,02	0,30	0,04	0,48						0,02			0,12	
Hentriacontane	2308	3,07	3100	3100	57	0,04	11,96	4,38	3,65	3,30	3,96	2,21	0,08	0,53	0,13	3,98	3,01	1,01	0,01	2,05	0,81	3,69	3,99	4,83	1,20	3,66	3,41	6,40	1,57	9,64	0,67	0,40	2,15	14,55	4,07
β-Sitosterol derivate[1]	2308	4,94	3100		43		0,07	0,04	0,18	0,11	0,04	0,05		0,12				0,05	0,05	0,02	0,05	0,02	0,04	0,04	0,06	0,03		0,05		0,03	0,04		0,07		0,03
Octacosan-1-ol	2316	3,92	3108	3118	57		0,03	20,67	18,97	20,13	12,11	26,94	1,02	2,28	29,56	22,20	12,26	0,04	0,03	11,80		11,25	25,81	19,57	19,94	12,92	18,69	20,24	29,72	13,13	15,11	14,94	25,93	22,24	23,28
Lupeol-H_2O[1]	2324	5,74	3116		55		0,03			0,02	0,01	0,01		0,01								0,04			0,02					0,03				0,01	

(8S,9S,10R,13R,14S,17R)-17-[(2R,5R)-5-Ethyl-6-methyl-heptan-2-yl]-10,13-dimethyl-2,7,8,9,11,12,14,15,16,17-decahydro-1H-cyclopenta[a]phenanthrene (Stigmasta-3,5-diene)	2332	5,33	3123	3172	81	0,14	0,35	0,32	0,15	0,38	0,08	0,18	0,12	0,61	0,04	0,21	0,05	0,14	0,15	0,05	0,09	0,25	0,07	0,16	0,35	0,23	0,17	0,15	0,05	0,14	0,16	0,17	0,17	0,11	0,13
Isoalkane[1]	2340	3,17	3131	3200	57	0,01		0,04	0,04				0,01	0,01	0,16		1,24			0,01		0,02	0,02		0,04	0,11				0,03			0,23		
Triacont-1-ene	2364	4,10	3154	3215	57			0,48	0,45	0,31		0,47	0,01	0,13	0,19		0,63			0,31	0,15	0,18		0,79						0,27				0,33	0,41
(2R)-2,5,7,8-Tetramethyl-2-[(4R,8R)-4,8,12-trimethyltridecyl]-3,4-dihydrochromen-6-ol (Vitamin E)	2364	5,86	3154	3130	165	7,10	9,50	12,64	9,76	13,06	5,23	12,48	3,83	1,87	2,83	9,76	0,46	1,33	0,87	2,15	2,76	5,63	15,97	4,11	0,71	2,04	9,39	10,39	5,78	3,64	2,38	2,67		6,01	3,66
(3S,8S,9S,10R,13R,14S,17R)-10,13-Dimethyl-17-[(2R)-6-methylheptan-2-yl]-2,3,4,7,8,9,11,12,14,15,16,17-dodeca-hydro-1H-cyclopenta[a]phenanthren-3-ol (Cholesterol)	2372	6,37	3162		43	0,16		0,09	0,09			0,02					0,02	0,06		0,01		0,08		0,05			0,07	0,03		0,08		0,09		0,13	0,07
1-Hexacosyl ether[1]	2396	2,79	3171		57																1,46														
Nonacosan-1-ol	2412	3,28	3200	3218[2]	57									0,08							0,07														
Dotriacontane	2412	3,60	3200	3200	57	0,05		0,05	0,04		0,77		0,02		0,67	0,04	0,65			0,06		0,02		0,06	0,64	0,23	0,10	0,03		0,05	0,03			0,10	0,03
(2R)-2,5,7,8-Tetramethyl-2-[(4R,8R)-4,8,12-trimethyltridecyl]-3,4-dihydrochromen-6-yl acetate (α-Tocopherol acetate)	2420	5,97	3206		165	0,23		0,02					0,01			0,13						0,02			0,13	0,02		0,02					8,24	0,07	0,05
Octacosyl acetate	2428	4,53	3213	3215	57	0,01	20,95	0,20	0,17	0,32	0,08	0,14	0,01	20,58	0,54	0,23	0,13	13,93	22,99	35,02	0,07	23,35	0,17	0,15	0,04	0,14		0,15		0,07	0,15	0,23	0,11	0,20	0,33
5-Nonadecylbenzene-1,3-diol	2444	7,95	3225		124	0,12		0,15	0,02			0,22	0,04			0,01	0,05	0,13				0,03		0,03	0,02	0,06			0,01	0,02	0,05	0,05		0,04	
Triacontane-1,30-diol	2468	5,01	3244		57			0,08	0,80					0,02	0,76	0,06	0,09	0,03	0,05		0,60	0,54		0,08	0,22		0,39			0,20	0,01	0,01	0,43	0,13	1,33
(3S,8S,9S,10R,13R,14S,17R)-17-[(2R,5R)-5,6-Dimethylheptan-2-yl]-10,13-dimethyl-2,3,4,7,8,9,11,12,14,15,16,17-dodecahydro-1H-cyclopenta[a]phenanthren-3-ol (Campesterol)	2492	7,84	3263		43	0,76		0,57	0,42			0,48	0,39									0,31		0,52	0,39	0,23	0,41	0,25			0,57	0,71		0,47	0,62
(3S,8S,9S,10R,13R,14S,17R)-17-[(E,2R,5S)-5-Ethyl-6-methylhept-3-en-2-yl]-10,13-dimethyl-2,3,4,7,8,9,11,12,14,15,16,17-dodecahydro-1H-cyclopenta[a]phenanthren-3-ol (Stigmasterol)	2548	0,24	3288		55	1,66		1,09			0,36	0,03	0,75			0,81	0,35	0,68	0,31	1,17		0,52	0,38	1,22	0,82	0,40	0,79	0,53		0,79	0,39	0,46		0,94	0,81
(E)-Tritriacont-3-ene	2548	3,87	3288		57			0,07								0,03						0,11								0,03				0,04	
Tritriacontane	2548	3,87	3288	3300	57		2,14	0,07	1,09	1,39		0,71	0,01		0,15			0,05							0,01								0,65		
Ethyl 2-phenyldocosanoate	2548	7,14	3288		104	0,04		0,39	0,04	0,41	0,22	0,16	0,37	0,16	0,12		0,04	0,12	0,28	0,08	0,43	0,21	0,05	0,05	0,10					0,11			0,16	0,12	0,08
Octacosanol ether[1]	2588	5,59	3288		57																									0,51					
Triacontan-1-ol	2612	5,60	3342	3318[2]	57																							0,59						1,11	0,74
pres. secondary alcohol[1]	2612	5,61	3342		57	0,03	0,02	0,02	0,02		0,05	0,24	0,01	0,02		0,02				0,31	0,01	0,01		0,79			0,59	0,59		0,45	0,01	0,03		0,04	0,03
(3S,9S,10R,13R,14R,17R)-17-[(E,2R,5S)-5-Ethyl-6-methylhept-3-en-2-yl]-10,13-dimethyl-2,3,4,9,11,12,14,15,16,17-decahydro-1H-cyclopenta[a]phenanthren-3-ol (Corbisterol)	2628	1,20	3342		43	2,26		2,06	0,26	0,24	0,20	3,58		0,41	0,21		0,74							0,34	1,66		1,38		0,33			3,74	0,13		
[(E,7R,11R)-3,7,11,15-Tetramethylhexadec-2-enyl], tetracosanoate (Phytyl tetracosanoate)	2628	5,82	3356		57		0,11	0,14	0,08	0,13	0,03	0,12		0,04	0,07	0,09	0,04	0,34		0,09	0,10	0,05	0,59	0,13	0,08	0,03	0,06	0,13	0,07	0,09	0,03	0,06		0,18	0,09
(3S,8S,9S,10R,13R,14S,17R)-10,13-dimethyl-17-[(E,2R)-5-Propan-2-ylhept-5-en-2-yl]-2,3,4,7,8,9,11,12,14,15,16,17-dodecahydro-1H-cyclopenta[a]phenanthren-3-ol (Fucosterol)	2644	2,02	3366		55	0,37		0,36	0,43			0,07				0,25							0,06	0,49		0,05				0,05	0,40	0,45		0,24	0,13
Octadecyl hexadecanoate	2644	5,94	3366		57	0,02	0,02					0,03	0,01								0,01	0,01	0,03	0,20								0,03	0,15		
(3S,4aR,6aR,6bS,8aR,12aR,14aR,14bR)-4,4,6a,6b,8a,11,11,14b-Octamethyl-1,2,3,4a,5,6,7,8,9,10,12,12a,14,14a-tetradecahydropicen-3-ol (β-Amyrin)	2668	3,20	3380	3337	218				0,05			0,02	0,07							0,04				0,09						0,10					
Triacontanoic acid	2676	3,91	3385		57		0,04																												
Hentriacontane-14,16-dione	2676	6,94	3385	3375	100	0,47	5,32	0,78	9,15	3,94	6,63	0,28	2,52	0,70	0,75	3,99	0,05	0,18		0,03	0,01	15,34	5,02	11,65	7,44	1,44	3,46	4,97	1,90	29,40	0,23		0,19	3,16	3,55
(1R,3aR,5aR,5bR,7aR,9S,11aR,11bR,13aR,13bR)-3a,5a,5b,8,8,11a-Hexamethyl-1-prop-1-en-2-yl-1,2,3,4,5,6,7,7a,9,10,11,11b,12,13,13a,13b-hexadecahydrocyclopenta[a]chrysen-9-ol (Lupeol)	2716	3,44	3409		69	0,31	0,01	0,14	0,07				0,02									0,08	0,03											0,04	
5-Heneicosylbenzene-1,3-diol	2764	4,08	3436		124	0,43		0,33	0,04			0,35	0,20			0,07	0,13	0,16				0,12		0,13	0,05	0,14				0,08	0,13	0,06		0,18	
(2R)-2,5,7,8-Tetramethyl-2-[(4R,8R)-4,8,12-trimethyltridecyl]-3,4-dihydrochromen-6-yl (α-Tocopherol mannoside)	2788	4,77	3449		165	2,87		0,48	0,16			0,24				0,10	0,07			0,02		0,10	0,05	0,17	0,28		0,17			0,11	0,18	0,19		0,09	0,10
Pentatriacontane	2884	6,21	3500	2500	57		0,06	0,04	0,04		0,15			0,02	0,15	0,07	0,15		0,02	0,05	0,02	0,04		0,04	0,21	0,08	0,05	0,07		0,05		0,04		0,10	0,03
Ethyl 2-phenyltetracosanoate	2924	2,74	3520		104	0,14	0,08	1,01	0,14	1,23	0,62	0,66	0,58	0,44	1,36		0,13	0,35	0,80	0,32	1,18	0,51	0,22	0,24	0,39		0,10	0,07	0,14	0,35		0,24	0,42	0,39	0,30
x-Hydroxyhentriacontane-14,16-dione[1]	3252	4,16	3633		100		0,07				0,14	0,20												0,17	0,17			0,18							
5-Tricosylbenzene-1,3-diol	3276	2,74	3636		124	0,21		0,14				0,12	0,22									0,18		0,16		0,19				0,08	0,12	0,06		0,27	

[1] The exact structure of the compound has not been established; [2]estimated by lit. RI of homologues; [3]determined for the synthesized compounds

Figure S1. The GC-MS chromatogram fragment of accession No. 1.

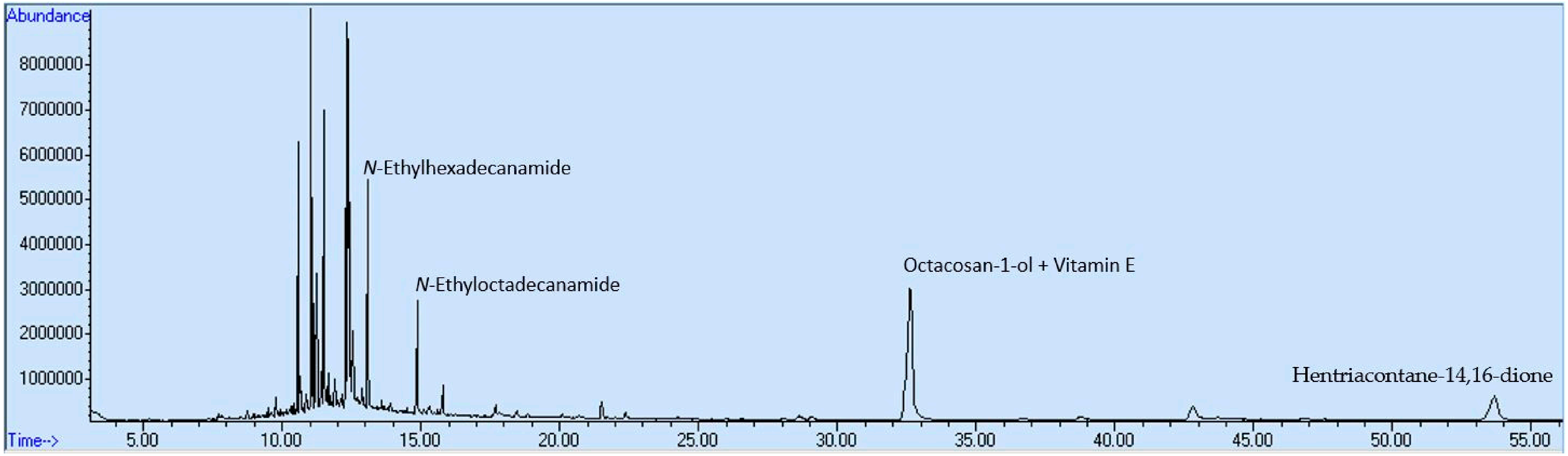

Abundance
8000000
7000000
6000000
5000000
4000000
3000000
2000000
1000000
N-Ethylhexadecanamide
N-Ethyloctadecanamide
Octacosan-1-ol + Vitamin E
Hentriacontane-14,16-dione
Time-->
5.00
10.00
15.00
20.00
25.00
30.00
35.00
40.00
45.00
50.00
55.00
